# Prioritizing patients for hip fracture surgery: the role of frailty and cardiac risk

**DOI:** 10.3389/fsurg.2024.1367457

**Published:** 2024-03-08

**Authors:** Maximilian Peter Forssten, Ahmad Mohammad Ismail, Ioannis Ioannidis, Marcelo A. F. Ribeiro, Yang Cao, Babak Sarani, Shahin Mohseni

**Affiliations:** ^1^ Department of Orthopedic Surgery, Faculty of Medicine and Health, Orebro University, Orebro, Sweden; ^2^ School of Medical Sciences, Orebro University, Orebro, Sweden; ^3^ Pontifical Catholic University of São Paulo, São Paulo, Brazil; ^4^ Khalifa University and Gulf Medical University, Abu Dhabi, United Arab Emirates; ^5^ Department of Surgery, Sheikh Shakhbout Medical City, Abu Dhabi, United Arab Emirates; ^6^ Clinical Epidemiology and Biostatistics, School of Medical Sciences, Faculty of Medicine and Health, Orebro University, Orebro, Sweden; ^7^ Division of Trauma and Acute Care Surgery, George Washington University School of Medicine & Health Sciences, Washington, DC, United States

**Keywords:** hip fracture, frailty, cardiac risk, surgical delay, mortality, surgical prioritization, risk stratification

## Abstract

**Introduction:**

The number of patients with hip fractures continues to rise as the average age of the population increases. Optimizing outcomes in this cohort is predicated on timely operative repair. The aim of this study was to determine if patients with hip fractures who are frail or have a higher cardiac risk suffer from an increased risk of in-hospital mortality when surgery is postponed >24 h.

**Methods:**

All patients registered in the 2013–2021 TQIP dataset who were ≥65 years old and underwent surgical fixation of an isolated hip fracture caused by a ground-level fall were included. Adjustment for confounding was performed using inverse probability weighting (IPW) while stratifying for frailty with the Orthopedic Frailty Score (OFS) and cardiac risk using the Revised Cardiac Risk Index (RCRI). The outcome was presented as the absolute risk difference in in-hospital mortality.

**Results:**

A total of 254,400 patients were included. After IPW, all confounders were balanced. A delay in surgery was associated with an increased risk of in-hospital mortality across all strata, and, as the degree of frailty and cardiac risk increased, so too did the risk of mortality. In patients with OFS ≥4, delaying surgery >24 h was associated with a 2.33 percentage point increase in the absolute mortality rate (95% CI: 0.57–4.09, *p* = 0.010), resulting in a number needed to harm (NNH) of 43. Furthermore, the absolute risk of mortality increased by 4.65 percentage points in patients with RCRI ≥4 who had their surgery delayed >24 h (95% CI: 0.90–8.40, *p* = 0.015), resulting in a NNH of 22. For patients with OFS 0 and RCRI 0, the corresponding NNHs when delaying surgery >24 h were 345 and 333, respectively.

**Conclusion:**

Delaying surgery beyond 24 h from admission increases the risk of mortality for all geriatric hip fracture patients. The magnitude of the negative impact increases with the patient's level of cardiac risk and frailty. Operative intervention should not be delayed based on frailty or cardiac risk.

## 1 Introduction

Roughly 2.7 million individuals experienced a hip fracture in 2010, accounting for ∼20% of all osteoporotic fractures in persons 50 years or older (1–3). It is expected that this figure will increase with the aging of the global population and the concomitant rise in life expectancy (4–8). This population is particularly susceptible to harm owing to their advanced age, high degree of frailty, and the substantial comorbidity burden present (9–16). Consequently, postoperative mortality rates remain elevated in the hip fracture population with incidences as high as 10%, 16%, and 27% being reported after 30 days, 90 days, and 1 year, respectively (16–18). As a result, hip fractures constitute a major burden on individuals, healthcare systems, and society as a whole, resulting in significant complications such as high mortality rates (19, 20), loss of functional ability (21–23), and prolonged hospital stays (24). These complications have substantial healthcare costs and require considerable resources from healthcare providers and public health systems (10, 12, 14, 25). Accordingly, the financial burden of hip fractures is significant, estimated at $6 billion annually in the United States alone (26).

While expediting surgical intervention has not been found to improve outcomes (27), numerous investigations have demonstrated an association between a delay in surgery and adverse outcomes in hip fracture patients (28–34). However, with a growing number of hip fracture patients and increasingly limited resources in healthcare (4–8, 35–38), achieving surgery within this timeframe for all patients might not always be feasible. Furthermore, frail patients and those with significant comorbid conditions are often subjected to preoperative testing for “clearance”, a process that may lead to delays in the timely execution of operative fixation. The aim of the current study was therefore to determine if hip fracture patients who are frailer, according to the Orthopedic Frailty Score, or have a higher cardiac risk, based on the Revised Cardiac Risk Index, suffer from a disproportionately increased risk of in-hospital mortality when surgery is postponed beyond 24 h of admission. The hypothesis was that frail patients and those with an elevated cardiac risk would exhibit a higher mortality rate than healthier patients when surgery is delayed more than 24 h.

## 2 Materials and methods

The American College of Surgeon Trauma Quality Improvement Project (TQIP) database was used to identify patients with isolated hip fractures from 2013 to 2021. Inclusion criteria included: age 65 years or older and undergoing surgical fixation after suffering an isolated hip fracture due to a ground-level fall. An isolated hip fracture was defined as a patient with a hip fracture and an abbreviated injury scale (AIS) ≤1 in all other regions. Patients were excluded if they underwent surgery >5 days after admission (30), if the time to surgery was missing, or if they had a lower extremity AIS of 6. Patient demographics (age, sex, race, comorbidities), clinical characteristics (AIS for all regions, type of fracture, type of surgery), and discharge disposition were abstracted. The need for ethical approval from an institutional review board was waived for this study, as all analyses were conducted using an anonymized, retrospective dataset. This study adheres to the ethical principles outlined in the Declaration of Helsinki and follows the reporting guidelines set forth by the Strengthening the Reporting of Observational Studies in Epidemiology (STROBE) guidelines (39).

### 2.1 Calculating the orthopedic frailty score

The Orthopedic Frailty Score (OFS) is a validated frailty score developed for predicting 30-day and 90-day mortality in hip fracture patients and has also been associated with an increased risk of in-hospital mortality, complications, failure-to-rescue, as well as a longer and more costly hospital stay (40, 41). The OFS was calculated based on the presence of 5 variables: an age ≥85 years old, non-independent functional status (i.e., requiring assistance with activities of daily life), institutionalization, congestive heart failure, and a history of malignancy (local or metastatic, excluding non-invasive skin cancer). For each variable present, patients received 1 point; the maximum possible score was 5 (40).

### 2.2 Calculating the revised cardiac risk index

In previous investigations, the Revised Cardiac Risk Index (RCRI) has been associated with an increased risk of mortality, up to 1 year, after hip fracture surgery (13, 15). According to the RCRI, patients receive 1 point each for the presence of high-risk surgery (any intraperitoneal, intrathoracic, and suprainguinal vascular procedure), history of cerebrovascular disease, renal insufficiency (defined as acute kidney injury or chronic kidney disease), diabetes mellitus, ischemic heart disease, as well as congestive heart failure (42–45). Hip fracture surgery is considered intermediate risk surgery by the American College of Cardiology and the American Heart Association guidelines; therefore, points for high-risk surgery were not awarded to any patient in this study (46). Accordingly, the maximum possible score was 5.

### 2.3 Statistical analysis

Patients were divided into two groups based on their time to surgery from admission: ≤24 h and >24 h. Patient demographics and other clinical features were summarized and compared to characterize differences between the groups. Categorical variables were expressed as counts along with their corresponding percentages. Continuous variables which exhibited a non-normal distribution, were presented using the median and interquartile range (IQR), while normally distributed variables were presented as a mean and standard deviation (SD). Statistical significance testing was conducted as follows: For categorical variables, either the Chi-square test or Fisher's exact test was employed to assess the significance of differences between the groups. On the other hand, for continuous variables, the Mann–Whitney *U*-test or Student's *t*-test was utilized. The primary endpoint was in-hospital mortality.

Patients were stratified based on their OFS (OFS 0, 1, 2, 3, ≥4) as well as their RCRI (RCRI 0, 1, 2, 3, ≥4). To minimize any differences in demographics and clinical characteristics between the cohorts within each stratum, the inverse probability weighting (IPW) method was employed. The probability of undergoing surgery >24 h after admission was determined using a logistic regression model. This model included age, sex, race, highest AIS in each region (head, face, neck, spine, thorax, abdomen, upper extremity, lower extremity, external), type of fracture, type of surgery, and comorbidities (hypertension, history of myocardial infarction, congestive heart failure, history of peripheral vascular disease, cerebrovascular disease, dementia, institutionalization, non-independent functional status, chronic obstructive pulmonary disease, smoking status, chronic renal failure, diabetes mellitus, cirrhosis, coagulopathy, currently receiving chemotherapy for cancer, metastatic cancer, drug use disorder, alcohol use disorder, major psychiatric illness, and the presence of advanced directives limiting care) as predictors. The weights were calculated as $\frac{1}{\mathrm{probabilty}\;\mathrm{of}\;\mathrm{undergoing}\;\mathrm{surgery}\;>24\;\mathrm{hours}\;\mathrm{after}\;\mathrm{admission}}$ for patients who underwent surgery >24 h after admission and $\frac{1}{1\text{-}\;\mathrm{probabilty}\;\mathrm{of}\;\mathrm{undergoing}\;\mathrm{surgery}\;\;>24\;\mathrm{hours}\;\mathrm{after}\;\mathrm{admission}}$ for patients who underwent surgery ≤24 h after admission. Balance after weighting was evaluated using absolute standardized differences (ASD). An ASD <0.1 was considered clinically balanced (47). The absolute risk difference (ARD) between patients who underwent surgery >24 h and ≤24 h from admission and corresponding 95% confidence intervals (CIs) were then calculated within each stratum (48).

Missing data was managed using multiple imputation by chained equations. The statistical analysis was performed with the statistical programming language R (R Foundation for Statistical Computing, Vienna, Austria) utilizing the packages tidyverse, haven, mice, parallel, and survey (49). Statistical significance was set *a priori* as a two-tailed *p*-value less than 0.05.

## 3 Results

**Figure 1:**
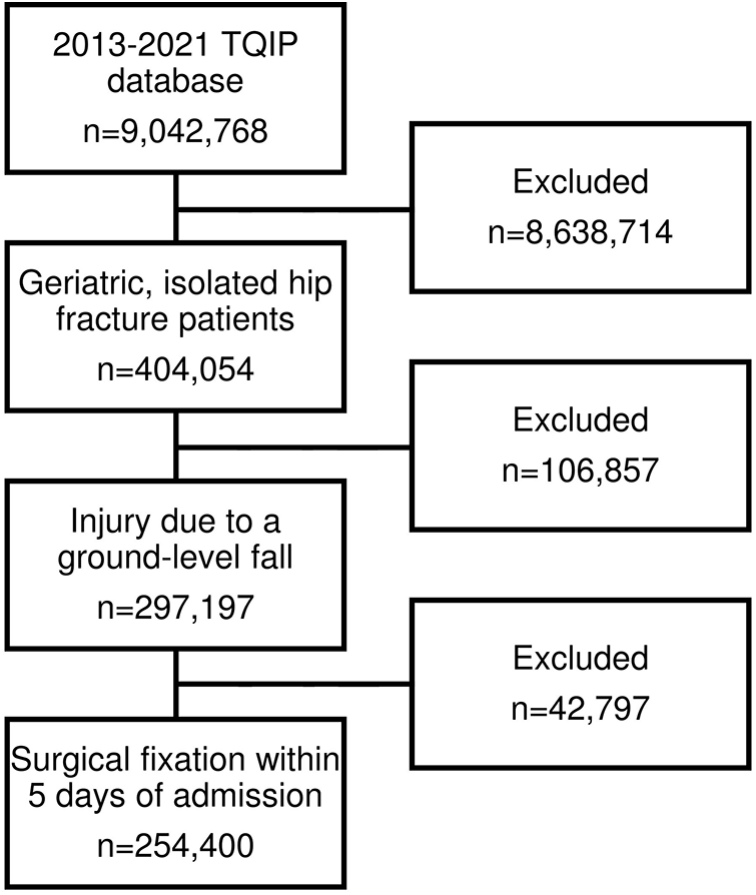
Flow chart describing selection of sample population.

After applying the inclusion and exclusion criteria, 254,400 patients were included for further analysis (Figure 1). Forty-one percent (*N* = 103,093) underwent surgery >24 h after admission. Those who underwent delayed surgery were more often male (34.1% vs. 29.4%, *p* < 0.001) as well as more likely to be Black (4.3% vs. 3.4%, *p* < 0.001) or Asian (1.3% vs. 1.1%, *p* < 0.001). Those who underwent delayed surgery were also more likely to be frail (OFS ≥2: 21.4% vs. 17.6%, *p* < 0.001) and suffer from an elevated cardiac risk (RCRI ≥2: 11.4% vs. 7.2%, *p* < 0.001). All comorbidities were more common in patients who underwent surgery >24 h after admission, except for being a current smoke and major psychiatric illness. Accordingly, the crude in-hospital mortality rate was higher among patients who underwent delayed surgery (1.9% vs. 1.2%, *p* < 0.001) (Table 1). There was no clinically significant difference in injury severity or vitals on admission. Cervical fractures were more common in those who underwent surgery >24 h after admission (38.7% vs. 34.9%, *p* < 0.001) while pertrochanteric fractures were less common (45.9% vs. 50.7%, *p* < 0.001). Accordingly, arthroplasty was performed more often in patients who underwent delayed surgery (38.9% vs. 32.6%, *p* < 0.001) (Table 2).

**Table 1:** Demographics of geriatric hip fracture patients.

	Surgery ≤24 h (*N* = 151,307)	Surgery >24 h (*N* = 103,093)	*P*-value
In-hospital mortality, *n* (%)	1,794 (1.2)	1,942 (1.9)	<0.001
Age, median [IQR]	80 [74–85]	81 [74–85]	<0.001
Sex, *n* (%)			<0.001
Female	106,615 (70.5)	67,778 (65.7)	
Male	44,461 (29.4)	35,163 (34.1)	
Missing	231 (0.2)	152 (0.1)	
Race, *n* (%)			
White	137,675 (91.0)	91,798 (89.0)	<0.001
Black	5,113 (3.4)	4,471 (4.3)	<0.001
Asian	1,623 (1.1)	1,337 (1.3)	<0.001
American Indian	977 (0.6)	455 (0.4)	<0.001
Pacific Islander	146 (0.1)	109 (0.1)	0.495
Other	4,228 (2.8)	3,630 (3.5)	<0.001
Missing	772 (0.5)	837 (0.8)	
OFS, *n* (%)			<0.001
0	74,081 (49.0)	44,508 (43.2)	
1	50,570 (33.4)	36,659 (35.6)	
2	20,934 (13.8)	16,857 (16.4)	
3	5,243 (3.5)	4,590 (4.5)	
≥4	479 (0.3)	479 (0.5)	
RCRI, *n* (%)			<0.001
0	99,168 (65.5)	59,969 (58.2)	
1	41,129 (27.2)	31,405 (30.5)	
2	9,214 (6.1)	9,464 (9.2)	
3	1,585 (1.0)	1,975 (1.9)	
≥4	211 (0.1)	280 (0.3)	
Hypertension, *n* (%)	100,191 (66.2)	71,472 (69.3)	<0.001
Previous myocardial infarction, *n* (%)	2,241 (1.5)	2,179 (2.1)	<0.001
Congestive heart failure, *n* (%)	12,644 (8.4)	14,042 (13.6)	<0.001
Peripheral vascular disease, *n* (%)	3,289 (2.2)	2,851 (2.8)	<0.001
Cerebrovascular disease, *n* (%)	8,798 (5.8)	7,385 (7.2)	<0.001
Dementia, *n* (%)	27,996 (18.5)	19,433 (18.8)	0.028
Institutionalized, *n* (%)	17,137 (11.3)	12,664 (12.3)	<0.001
Non-independent functional status, *n* (%)	35,584 (23.5)	26,918 (26.1)	<0.001
COPD, *n* (%)	23,526 (15.5)	18,504 (17.9)	<0.001
Current smoker, *n* (%)	15,988 (10.6)	10,471 (10.2)	<0.001
Chronic renal failure, *n* (%)	5,046 (3.3)	5,148 (5.0)	<0.001
Diabetes mellitus, *n* (%)	34,610 (22.9)	26,581 (25.8)	<0.001
Cirrhosis, *n* (%)	1,265 (0.8)	1,157 (1.1)	<0.001
Coagulopathy, *n* (%)	5,716 (3.8)	7,417 (7.2)	<0.001
Currently receiving chemotherapy for cancer, *n* (%)	1,521 (1.0)	1,168 (1.1)	0.002
Metastatic cancer, *n* (%)	1,963 (1.3)	1,583 (1.5)	<0.001
Drug use disorder, *n* (%)	1,327 (0.9)	1,049 (1.0)	<0.001
Alcohol use disorder, *n* (%)	3,456 (2.3)	2,574 (2.5)	<0.001
Major psychiatric illness, *n* (%)	18,670 (12.3)	12,184 (11.8)	<0.001
Advanced directive limiting care, *n* (%)	13,462 (8.9)	9,639 (9.3)	<0.001

OFS, orthopedic frailty score; RCRI, revised cardiac risk index; COPD, chronic obstructive pulmonary disease.

**Table 2:** Clinical characteristics of geriatric hip fracture patients.

	Surgery ≤24 h (*N* = 151,307)	Surgery >24 h (*N* = 103,093)	*P*-value
Head AIS, *n* (%)			<0.001
Injury not present	146,865 (97.1)	99,578 (96.6)	
1	4,442 (2.9)	3,515 (3.4)	
Face AIS, *n* (%)			<0.001
Injury not present	147,612 (97.6)	100,263 (97.3)	
1	3,695 (2.4)	2,830 (2.7)	
Neck AIS, *n* (%)			0.991
Injury not present	151,213 (99.9)	103,028 (99.9)	
1	94 (0.1)	65 (0.1)	
Spine AIS, *n* (%)			0.030
Injury not present	151,170 (99.9)	102,970 (99.9)	
1	137 (0.1)	123 (0.1)	
Thorax AIS, *n* (%)			<0.001
Injury not present	150,468 (99.4)	102,383 (99.3)	
1	839 (0.6)	710 (0.7)	
Abdomen AIS, *n* (%)			<0.001
Injury not present	150,975 (99.8)	102,731 (99.6)	
1	332 (0.2)	362 (0.4)	
Upper extremity AIS, *n* (%)			0.025
Injury not present	139,932 (92.5)	95,094 (92.2)	
1	11,375 (7.5)	7,999 (7.8)	
Lower extremity AIS, *n* (%)			0.054
3	151,294 (100.0)	103,076 (100.0)	
4	11 (0.0)	16 (0.0)	
5	1 (0.0)	0 (0.0)	
Missing	1 (0.0)	1 (0.0)	
External AIS, *n* (%)			<0.001
Injury not present	150,420 (99.4)	102,306 (99.2)	
1	887 (0.6)	787 (0.8)	
Systolic blood pressure, mean (SD)	151 (±27.3)	149 (±28.0)	<0.001
Missing, *n* (%)	5581 (3.7)	3699 (3.6)	
Pulse rate, mean (SD)	80.5 (±15.1)	81.1 (±16.2)	<0.001
Missing, *n* (%)	5464 (3.6)	3640 (3.5)	
Temperature, mean (SD)	36.7 (±0.6)	36.7 (±0.7)	<0.001
Missing, *n* (%)	10,692 (7.1)	7,345 (7.1)	
Oxygen saturation, median [IQR]	97 [95–98]	97 [94–98]	0.163
Missing, *n* (%)	7,576 (5.0)	5,290 (5.1)	
Respiratory rate, mean (SD)	17.8 (±3.0)	18.0 (±3.2)	<0.001
Missing, *n* (%)	6,674 (4.4)	4,324 (4.2)	
Type of fracture, *n* (%)			<0.001
Cervical	52,879 (34.9)	39,946 (38.7)	
Basicervical	7,838 (5.2)	5,600 (5.4)	
Pertrochanteric	76,657 (50.7)	47,306 (45.9)	
Subtrochanteric	5,195 (3.4)	3,252 (3.2)	
Missing	8,738 (5.8)	6,989 (6.8)	
Type of surgery, *n* (%)			<0.001
Internal fixation	101,964 (67.4)	62,996 (61.1)	
Arthroplasty	49,343 (32.6)	40,097 (38.9)	

Systolic blood pressure is measured in mmHg. Temperature is measured in degrees Celsius.

AIS, abbreviated injury scale.

**Figure 2:**
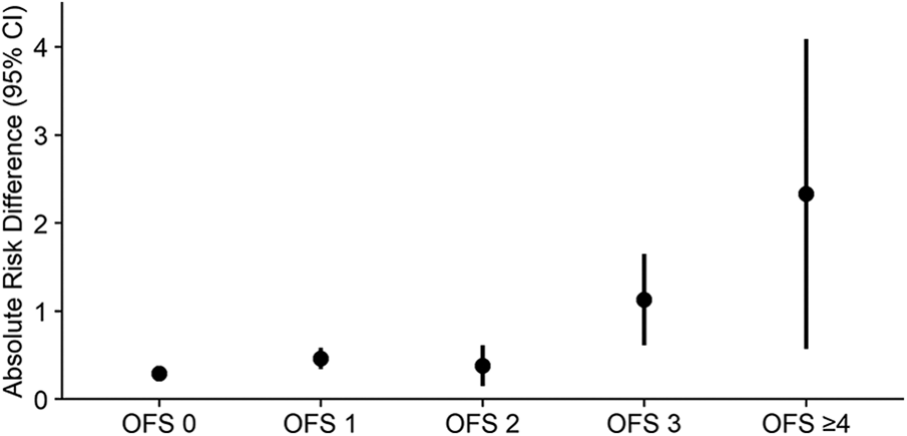
Difference in absolute risk of in-hospital mortality in hip fracture patients after IPW, stratified by frailty. IPW, inverse probability weighting; CI, confidence interval; OFS, orthopedic frailty score.

Balance was achieved after IPW, with all included potential confounders exhibiting an ASD <0.1 (Supplementary Tables S1–S10). A delay in hip fracture surgery by >24 h was associated with an increased risk of in-hospital mortality, irrespective of the degree of frailty. Nevertheless, the adjusted ARD increased along with the OFS [Spearman's *ρ* (95% CI): 0.85 (0.60–1.00), *p* < 0.001]. Compared to patients who underwent surgery within 24 h, surgery >24 h after admission was associated with a 0.29 percentage point increase in the mortality rate among patients with OFS 0 [adjusted ARD (95% CI): 0.29 (0.22–0.37), *p* < 0.001] (Table 3), which corresponds to a number needed to harm (NNH) of 345 (50). On the other hand, in patients with OFS ≥4, delaying surgery >24 h was associated with a 2.33 percentage point increase in the mortality rate [adjusted ARD (95% CI): 2.33 (0.57–4.09), *p* = 0.010] (Table 3 and Figure 2), resulting in a NNH of 43.

**Table 3:** Difference in absolute risk of in-hospital mortality in hip fracture patients after IPW, stratified by frailty.

Level of frailty	Surgery ≤24 h (%)	Surgery >24 h (%)	ARD (95% CI)	*P*-value
OFS 0	0.7	1.0	0.29 (0.22–0.37)	<0.001
OFS 1	1.4	1.9	0.46 (0.34–0.58)	<0.001
OFS 2	2.4	2.8	0.38 (0.15–0.61)	0.001
OFS 3	3.0	4.1	1.13 (0.61–1.65)	<0.001
OFS ≥4	2.9	5.2	2.33 (0.57–4.09)	0.010

Using IPW, all strata were balanced in regard to age, sex, race, highest AIS in each region, type of fracture, type of surgery, and comorbidities.

IPW, inverse probability weighting; ARD, absolute risk difference; CI, confidence interval; OFS, orthopedic frailty score; AIS, abbreviated injury scale.

**Figure 3:**
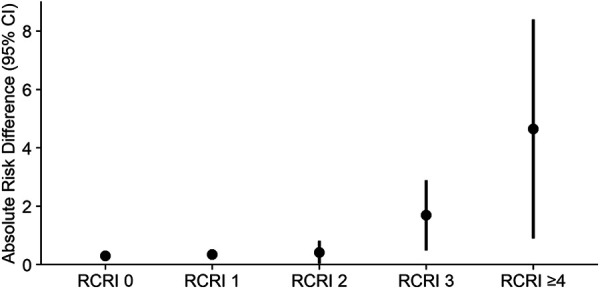
Difference in absolute risk of in-hospital mortality in hip fracture patients after IPW, stratified by cardiac risk. IPW, inverse probability weighting; CI, confidence interval; RCRI, revised cardiac risk index.

Similar results were observed when patients were stratified according to their RCRI. A delay in hip fracture surgery by >24 h was associated with an increased risk of in-hospital mortality, irrespective of the degree of cardiac risk, and the risk of mortality increased along with the RCRI [Spearman's *ρ* (95% CI): 0.87 (0.60–1.00), *p* < 0.001]. In patients with RCRI 0, delaying surgery >24 h was associated with a 0.30 percentage point increase in the in-hospital mortality rate [adjusted ARD (95% CI): 0.30 (0.24–0.37), *p* < 0.001] (Table 4), compared to patients who underwent surgery within 24 h of admission; this corresponds to a NNH of 333. Conversely, the risk of mortality increased by 4.65 percentage points in patients with RCRI ≥4 who had their surgery delayed >24 h [adjusted ARD (95% CI): 4.65 (0.90–8.40), *p* = 0.015] (Table 4 and Figure 3), resulting in a NNH of 22.

**Table 4:** Difference in absolute risk of in-hospital mortality in hip fracture patients after IPW, stratified by cardiac risk.

Level of cardiac risk	Surgery ≤24 h (%)	Surgery >24 h (%)	ARD (95% CI)	*P*-value
RCRI 0	0.8	1.1	0.30 (0.24–0.37)	<0.001
RCRI 1	1.6	1.9	0.35 (0.22–0.49)	<0.001
RCRI 2	3.5	3.9	0.42 (0.04–0.81)	0.030
RCRI 3	6.3	8.0	1.69 (0.49–2.89)	0.006
RCRI ≥4	7.7	12.3	4.65 (0.90–8.40)	0.015

Using IPW, all strata were balanced in regard to age, sex, race, highest AIS in each region, type of fracture, type of surgery, and comorbidities.

IPW, inverse probability weighting; ARD, absolute risk difference; CI, confidence interval; RCRI, revised cardiac risk index; AIS, abbreviated injury scale.

## 4 Discussion

In this analysis based on 254,400 patients recorded in the TQIP registry it was found that delaying hip fracture surgery by more than 24 h was linked to an elevated risk of in-hospital mortality, regardless of the patient's level of frailty or cardiac risk. However, the absolute risk of mortality increased along with a patients' level of frailty and cardiac risk. At the highest levels, postponing surgery in patients with an OFS ≥4 led to a NNH of 43, whereas a similar delay in patients with an RCRI ≥4 resulted in a NNH of 22. These findings underscore the importance of timely hip fracture surgery in reducing in-hospital mortality risk, particularly for patients with higher degrees of frailty or cardiac risk.

Several studies have analyzed the association between a delay in surgical fixation of hip fractures and adverse outcomes. While some have failed to detect a significant association with adverse outcomes and the HIP ATTACK study did not find any advantage to accelerating surgery (27, 51, 52), the majority do find that a delay in definitive fixation is associated with an increased risk of mortality as well as other unfavorable outcomes (28–34), which is in line with the current analyses. Two previous systematic reviews, one by Khan et al. with 291,413 patients and a second by Klestil et al. with 31,242 patients, have detected an association between a delay beyond 48 h and an increased risk of mortality, complications, and a longer hospital stay (28, 29). A large American study be Tran et al. comprising almost 2 million estimated patients also found that the same delay resulted in an increased risk of in-hospital mortality, complications, and non-home discharge, as well as that the risk of these adverse outcomes increased along with the duration of the delay (30). Leer-Salvesen et al. found that this association also extended up to 1 year after surgery, even after controlling for differences in anticoagulant use (32). Pincus et al. published a thorough analysis of the subject that identified 24 h as the optimal cutoff for early vs. delayed surgery based on restricted cubic splines. They too found that delayed surgery was associated with an increased risk of mortality and complications. Furthermore, this association remained even after limiting the analysis to patients without comorbidity and those receiving surgery within 36 h, for whom confounding by indication should not play a role (31). Finally, two studies by Greve et al, that also used a 24 h cutoff, found that delaying hip fracture surgery increased the risk of mortality and specific complications. However, in a similar vein to the current investigation, this association was only detected in those who had an American Society of Anesthesiologists Classification of 3 or 4 (33, 34).

While administrative or resource limitations have been responsible for up to two-thirds of delays in definitive hip fracture fixation (53), it is important to explore other potential reasons behind this observed association. One factor to consider is that patients undergoing delayed surgery suffered from a higher comorbidity burden or were more severely injured. Although patients who underwent surgery >24 h after admission exhibited a higher comorbidity burden in the current study, both comorbidities and injury severity were effectively balanced through IPW. This suggests that these factors alone cannot account for the observed relationship. Another commonly cited cause may be delay due to preadmission anticoagulant and antithrombotic therapy. However, even after adjusting for these therapies, the association between surgical delay and adverse outcomes persists (31, 32, 34). Furthermore, while anticoagulant therapy was not specifically adjusted for in the current analysis, the rate of anticoagulant use was balanced after IPW in all strata. On the other hand, some may argue that the association could be due to necessary delays related to preoperative complications, testing, or the need for preoperative optimization. Nevertheless, this was also considered in the investigation by Pincus et al., who even observed the association between surgical delay and adverse outcomes among patients who were operated within 36 h, a time period which was selected to exclude delays due to preoperative complications and still be more than sufficient to optimize the patient (31).

An alternative hypothesis should therefore be considered when considering the trends observed in the current investigation. According to the current results as well as the results of Greve et al., the patients who suffer the most from a postponement of hip fracture surgery are those that are the most frail, have the highest cardiac risk, and are the least fit-for-surgery (33, 34). It could consequently be hypothesized that these patients may be less able to endure the prolonged period of physiological stress resulting from a delay in definitive fixation. When individuals suffer a hip fracture, it triggers a surge in catecholamines and cortisol, which can harm multiple organ systems (54, 55). Frail patients, who by definition struggle to maintain homeostasis in response to external stressors (56–59), are likely to be particularly vulnerable to extended periods of physiological stress. Similarly, individuals with elevated cardiac risk factors may also face a heightened risk of decompensation when subjected to this prolonged stress.

Ideally, hip fracture surgery should never be delayed longer than absolutely necessary, taking into account both humane considerations and the potential impact on mortality. However, it is important to recognize that healthcare resources are finite and are expected to become even more limited over time (35–38). This, coupled with the increasing prevalence of hip fractures worldwide (4–8), means that not all patients can be operated within the 24-h cutoff. Given that patients with a higher OFS and RCRI seem to be the most adversely affected by delays in hip fracture surgery, it may be justifiable to prioritize frailer patients and those with an elevated cardiac risk over those who are relatively healthier. The OFS and RCRI stand out as excellent tools for this purpose due to their simplicity, each relying on just 5 dichotomous variables that are easily accessible from patients' medical records. Nonetheless, it is crucial to emphasize that these scores are simplifications, and individual patients' clinical profiles should always be carefully considered. Our results also suggest that preoperative testing and optimization in those with a higher OFS and RCRI score should be prioritized so as to enable operation within 24 h of admission.

This study benefits from large sample size, including over 250,000 patients from the largest trauma registry in the United States. Furthermore, more than 30 potential confounders could be adjusted for in the analysis, spanning demographic and clinical characteristics, as well as comorbidities. Nonetheless, there are also limitations that bear consideration. Of particular note, the cause of delay is not registered in the TQIP registry; however, even when this has been accounted for in previous investigations, the association between surgical delay and adverse outcomes remains (31, 53). Another crucial aspect to bear in mind is that this analysis does not address the prioritization of different types of fractures. For instance, it does not provide guidance on whether a pelvic fracture with an OFS of 4 should take precedence over a hip fracture with an OFS of 1. Finally, this study has the same limitations as all retrospective cohort studies, including the risk of residual confounding, inability to prove causation, as well as reliance on the accuracy of the underlying dataset. However, it would be challenging ethically to investigate the effect of delayed surgery with any other study design.

### 4.1 Conclusion

Delaying surgery beyond 24 h from admission increases the risk of mortality for all geriatric hip fracture patients; however, the magnitude of the negative impact increases with the patient's level of cardiac risk and frailty. The Orthopedic Frailty Score and Revised Cardiac Risk Index could therefore potentially be used to aid in prioritizing patients for hip fracture surgery when resources are limited. Nevertheless, care should also be taken to consider the patients' complete clinical profiles before making a final decision.

## Data Availability

The raw data supporting the conclusions of this article will be made available by the authors, without undue reservation.

## References

[1] Rapp K Büchele G Dreinhöfer K Bücking B Becker C Benzinger P. Epidemiology of hip fractures. Z Gerontol Geriat. (2019) 52:10–6. 10.1007/s00391-018-1382-z PMC6353815 29594444

[2] Oden A Dawson A Dere W Johnell O Jonsson B Kanis JA. Lifetime risk of hip fractures is underestimated. Osteoporos Int. (1998) 8:599–603. 10.1007/s001980050105 10326067

[3] Johnell O Kanis JA. An estimate of the worldwide prevalence and disability associated with osteoporotic fractures. Osteoporos Int. (2006) 17:1726–33. 10.1007/s00198-006-0172-4 16983459

[4] Sing C-W Lin T-C Bartholomew S Bell JS Bennett C Beyene K Global epidemiology of hip fractures: a study protocol using a common analytical platform among multiple countries. BMJ Open. (2021) 11:e047258. 10.1136/bmjopen-2020-047258 34321298 PMC8319985

[5] Study Finds Global Hip Fractures Expected to Double by 2050. Endocrinology Network. (2022). Available online at: https://www.endocrinologynetwork.com/view/top-news-in-endocrinology-for-2022 (Accessed January 31, 2023).

[6] Rosengren BE Björk J Cooper C Abrahamsen B. Recent hip fracture trends in Sweden and Denmark with age-period-cohort effects. Osteoporos Int. (2017) 28:139–49. 10.1007/s00198-016-3768-3 27647528 PMC5206266

[7] Reginster J-Y Burlet N. Osteoporosis: a still increasing prevalence. Bone. (2006) 38:S4–9. 10.1016/j.bone.2005.11.024 16455317

[8] Gullberg B Johnell O Kanis JA. World-wide projections for hip fracture. Osteoporos Int. (1997) 7:407–13. 10.1007/pl00004148 9425497

[9] Forssten MP Mohammad Ismail A Ioannidis I Wretenberg P Borg T Cao Y The mortality burden of frailty in hip fracture patients: a nationwide retrospective study of cause-specific mortality. Eur J Trauma Emerg Surg. (2022) 49:1467–75. 10.1007/s00068-022-02204-6 36571633 PMC10229683

[10] Williamson S Landeiro F McConnell T Fulford-Smith L Javaid MK Judge A Costs of fragility hip fractures globally: a systematic review and meta-regression analysis. Osteoporos Int. (2017) 28:2791–800. 10.1007/s00198-017-4153-6 28748387

[11] van de Ree CLP Landers MJF Kruithof N Munter L de Slaets JPJ Gosens T Effect of frailty on quality of life in elderly patients after hip fracture: a longitudinal study. BMJ Open. (2019) 9:e025941. 10.1136/bmjopen-2018-025941 31324679 PMC6661564

[12] Wong BLL Chan YH O’Neill GK Murphy D Merchant RA. Frailty, length of stay and cost in hip fracture patients. Osteoporos Int. (2023) 34:59–68. 10.1007/s00198-022-06553-1 36197493

[13] Forssten MP Mohammad Ismail A Borg T Ahl R Wretenberg P Cao Y Postoperative mortality in hip fracture patients stratified by the revised cardiac risk index: a Swedish nationwide retrospective cohort study. Trauma Surg Acute Care Open. (2021) 6:e000778. 10.1136/tsaco-2021-000778 34395919 PMC8314694

[14] Ferris H Brent L Sorensen J. Cost of hospitalisation for hip fracture-findings from the Irish hip fracture database. Osteoporos Int. (2022) 33:1057–65. 10.1007/s00198-021-06294-7 35015086 PMC8749353

[15] Forssten MP Mohammad Ismail A Sjolin G Ahl R Wretenberg P Borg T The association between the revised cardiac risk index and short-term mortality after hip fracture surgery. Eur J Trauma Emerg Surg. (2022) 48:1885–92. 10.1007/s00068-020-01488-w 32944823 PMC9192369

[16] Gundel O Thygesen LC Gögenur I Ekeloef S. Postoperative mortality after a hip fracture over a 15-year period in Denmark: a national register study. Acta Orthop. (2020) 91:58–62. 10.1080/17453674.2019.1680485 31635502 PMC7006693

[17] Abrahamsen B van Staa T Ariely R Olson M Cooper C. Excess mortality following hip fracture: a systematic epidemiological review. Osteoporos Int. (2009) 20:1633–50. 10.1007/s00198-009-0920-3 19421703

[18] NHFD 2019 annual report. Available online at: https://www.nhfd.co.uk/20/hipfractureR.nsf/docs/2019Report (Accessed October 18, 2020).

[19] Katsoulis M Benetou V Karapetyan T Feskanich D Grodstein F Pettersson-Kymmer U Excess mortality after hip fracture in elderly persons from Europe and the USA: the CHANCES project. J Intern Med. (2017) 281:300–10. 10.1111/joim.12586 28093824

[20] Panula J Pihlajamäki H Mattila VM Jaatinen P Vahlberg T Aarnio P Mortality and cause of death in hip fracture patients aged 65 or older—a population-based study. BMC Musculoskelet Disord. (2011) 12:105. 10.1186/1471-2474-12-105 21599967 PMC3118151

[21] Ouellet JA Ouellet GM Romegialli AM Hirsch M Berardi L Ramsey CM Functional outcomes after hip fracture in independent community-dwelling patients. J Am Geriatr Soc. (2019) 67:1386–92. 10.1111/jgs.15870 30964203 PMC6941577

[22] Dyer SM Crotty M Fairhall N Magaziner J Beaupre LA Cameron ID Fragility fracture network (FFN) rehabilitation research special interest group. A critical review of the long-term disability outcomes following hip fracture. BMC Geriatr. (2016) 16:158. 10.1186/s12877-016-0332-0 27590604 PMC5010762

[23] Marottoli RA Berkman LF Cooney LM. Decline in physical function following hip fracture. J Am Geriatr Soc. (1992) 40:861–6. 10.1111/j.1532-5415.1992.tb01980.x 1512379

[24] Nikkel LE Kates SL Schreck M Maceroli M Mahmood B Elfar JC. Length of hospital stay after hip fracture and risk of early mortality after discharge in New York state: retrospective cohort study. Br Med J. (2015) 351:h6246. 10.1136/bmj.h6246 26655876 PMC4674667

[25] Leal J Gray AM Prieto-Alhambra D Arden NK Cooper C Javaid MK Impact of hip fracture on hospital care costs: a population-based study. Osteoporos Int. (2016) 27:549–58. 10.1007/s00198-015-3277-9 26286626 PMC4740562

[26] Adeyemi A Delhougne G. Incidence and economic burden of intertrochanteric fracture. JB JS Open Access. (2019) 4:e0045. 10.2106/JBJS.OA.18.00045 31161153 PMC6510469

[27] Borges FK Bhandari M Guerra-Farfan E Patel A Sigamani A Umer M Accelerated surgery versus standard care in hip fracture (HIP ATTACK): an international, randomised, controlled trial. Lancet (2020) 395:698–708. 10.1016/S0140-6736(20)30058-1 32050090

[28] Khan SK Kalra S Khanna A Thiruvengada MM Parker MJ. Timing of surgery for hip fractures: a systematic review of 52 published studies involving 291,413 patients. Injury. (2009) 40:692–7. 10.1016/j.injury.2009.01.010 19450802

[29] Klestil T Röder C Stotter C Winkler B Nehrer S Lutz M Impact of timing of surgery in elderly hip fracture patients: a systematic review and meta-analysis. Sci Rep. (2018) 8:13933. 10.1038/s41598-018-32098-7 30224765 PMC6141544

[30] Tran Z Hsiue PP Pan C Verma A Rahimtoola R Stavrakis A Impact of delayed intervention on clinical outcomes following traumatic hip fracture in the elderly: a national analysis. J Orthop. (2021) 27:74–8. 10.1016/j.jor.2021.09.006 34566352 PMC8449020

[31] Pincus D Ravi B Wasserstein D Huang A Paterson JM Nathens AB Association between wait time and 30-day mortality in adults undergoing hip fracture surgery. JAMA. (2017) 318:1994–2003. 10.1001/jama.2017.17606 29183076 PMC5820694

[32] Leer-Salvesen S Engesæter LB Dybvik E Furnes O Kristensen TB Gjertsen J-E. Does time from fracture to surgery affect mortality and intraoperative medical complications for hip fracture patients? An observational study of 73 557 patients reported to the Norwegian hip fracture register. Bone Joint J. (2019) 101-B:1129–37. 10.1302/0301-620X.101B9.BJJ-2019-0295.R1 31474142

[33] Greve K Modig K Talbäck M Bartha E Hedström M. No association between waiting time to surgery and mortality for healthier patients with hip fracture: a nationwide Swedish cohort of 59,675 patients. Acta Orthop. (2020) 91:1–6. 10.1080/17453674.2020.1754645 PMC8023952 32326789

[34] Greve K Ek S Bartha E Modig K Hedström M. Waiting more than 24 hours for hip fracture surgery is associated with increased risk of adverse outcomes for sicker patients: a nationwide cohort study of 63,998 patients using the Swedish hip fracture register. Acta Orthop. (2023) 94:87–96. 10.2340/17453674.2023.9595 36847752 PMC9972166

[35] Hurst SA Forde R Reiter-Theil S Slowther A-M Perrier A Pegoraro R Physicians’ views on resource availability and equity in four European health care systems. BMC Health Serv Res. (2007) 7:137. 10.1186/1472-6963-7-137 17764556 PMC1995213

[36] Directorate-General for Employment Social Affairs and Inclusion (European Commission), McGrath J. Analysis of Shortage and Surplus Occupations 2020. Brussels: LU: Publications Office of the European Union (2020). Available online at: https://data.europa.eu/doi/10.2767/933528 (Accessed October 22, 2023).

[37] AAMC Report Reinforces Mounting Physician Shortage. Washington, DC: AAMC (2021). Available online at: https://www.aamc.org/news-insights/press-releases/aamc-report-reinforces-mounting-physician-shortage (Accessed August 10, 2022).

[38] American Association of Colleges of Nursing. *Nursing shortage fact sheet. (Factsheet)*. (2023). Available online at: https://www.aacnnursing.org/news-data/fact-sheets/nursing-shortage (accessed October 22, 2023).

[39] *WMA—The World Medical Association-WMA declaration of Helsinki—ethical principles for medical research involving human subjects*. Available online at: https://www.wma.net/policies-post/wma-declaration-of-helsinki-ethical-principles-for-medical-research-involving-human-subjects/ (Accessed May 13, 2020). 10.1191/0969733002ne486xx 16010903

[40] Forssten MP Cao Y Trivedi DJ Ekestubbe L Borg T Bass GA Developing and validating a scoring system for measuring frailty in patients with hip fracture: a novel model for predicting short-term postoperative mortality. Trauma Surg Acute Care Open. (2022) 7:e000962. 10.1136/tsaco-2022-000962 36117728 PMC9472206

[41] Forssten MP Cao Y Mohammad Ismail A Ioannidis I Tennakoon L Spain DA Validation of the orthopedic frailty score for measuring frailty in hip fracture patients: a cohort study based on the United States national inpatient sample. Eur J Trauma Emerg Surg. (2023) 49:2155–63. 10.1007/s00068-023-02308-7 37349513 PMC10520138

[42] Lee TH Marcantonio ER Mangione CM Thomas EJ Polanczyk CA Cook EF Derivation and prospective validation of a simple index for prediction of cardiac risk of major noncardiac surgery. Circulation. (1999) 100:1043–9. 10.1161/01.cir.100.10.1043 10477528

[43] Lindenauer PK Pekow P Wang K Mamidi DK Gutierrez B Benjamin EM. Perioperative beta-blocker therapy and mortality after major noncardiac surgery. N Engl J Med. (2005) 353:349–61. 10.1056/NEJMoa041895 16049209

[44] Bass GA Duffy CC Kaplan LJ Sarani B Martin ND Mohammad Ismail A The revised cardiac risk index is associated with morbidity and mortality independent of injury severity in elderly patients with rib fractures. Injury. (2022) 54(1):56–62. 10.1016/j.injury.2022.11.039 36402584

[45] Forssten MP Bass GA Scheufler K-M Mohammad Ismail A Cao Y Martin ND Mortality risk stratification in isolated severe traumatic brain injury using the revised cardiac risk index. Eur J Trauma Emerg Surg. (2022) 48:4481–8. 10.1007/s00068-021-01841-7 34839374 PMC9712303

[46] Fleisher LA Beckman JA Brown KA Calkins H Chaikof E Fleischmann KE ACC/AHA 2007 guidelines on perioperative cardiovascular evaluation and care for noncardiac surgery: a report of the American college of cardiology/American heart association task force on practice guidelines (writing committee to revise the 2002 guidelines on perioperative cardiovascular evaluation for noncardiac surgery): developed in collaboration with the American society of echocardiography, American society of nuclear cardiology, heart rhythm society, society of cardiovascular anesthesiologists, society for cardiovascular angiography and interventions, society for vascular medicine and biology, and society for vascular surgery. Circulation. (2007) 116:e418–99. 10.1161/CIRCULATIONAHA.107.185699 17901357

[47] Austin PC. Using the standardized difference to compare the prevalence of a binary variable between two groups in observational research. Commun Stat Simul Comput. (2009) 38:1228–34. 10.1080/03610910902859574

[48] Hildebrandt M Bender R Gehrmann U Blettner M. Calculating confidence intervals for impact numbers. BMC Med Res Methodol. (2006) 6:32. 10.1186/1471-2288-6-32 16836748 PMC1569862

[49] R Development Core Team. *R: a language and environment for statistical computing*. (2008). Available online at: http://www.R-project.org/

[50] Suchmacher M Geller M. Chapter 2—determination of association strength between an exposure factor and an event in observational studies. In: Suchmacher M Geller M, editors. Practical Biostatistics. San Diego: Academic Press (2012). p. 19–29. 10.1016/B978-0-12-415794-1.00002-1

[51] Rijckevorsel VAJIM van Jong L de Verhofstad MHJ Roukema GR. Influence of time to surgery on clinical outcomes in elderly hip fracture patients: an assessment of surgical postponement due to non-medical reasons. Bone Joint J (2022) 104-B:1369–78. 10.1302/0301-620X.104B12.BJJ-2022-0172.R2 36453044 PMC9680196

[52] Majumdar SR Beaupre LA Johnston DWC Dick DA Cinats JG Jiang HX. Lack of association between mortality and timing of surgical fixation in elderly patients with hip fracture: results of a retrospective population-based cohort study. Med Care. (2006) 44:552–9. 10.1097/01.mlr.0000215812.13720.2e 16708004

[53] Al-Ani AN Samuelsson B Tidermark J Norling Å Ekström W Cederholm T Early operation on patients with a hip fracture improved the ability to return to independent living: a prospective study of 850 patients. J Bone Joint Surg Am. (2008) 90:1436. 10.2106/JBJS.G.00890 18594090

[54] Desborough JP. The stress response to trauma and surgery. Br J Anaesth. (2000) 85:109–17. 10.1093/bja/85.1.109 10927999

[55] Moor D Aggarwal G Quiney N. Systemic response to surgery. Surg Oxf Int Ed. (2017) 35:220–3. 10.1016/j.mpsur.2017.01.013

[56] Fried LP Tangen CM Walston J Newman AB Hirsch C Gottdiener J Frailty in older adults: evidence for a phenotype. J Gerontol A Biol Sci Med Sci. (2001) 56:M146–56. 10.1093/gerona/56.3.m146 11253156

[57] Clegg A Young J Iliffe S Rikkert MO Rockwood K. Frailty in elderly people. Lancet. (2013) 381:752–62. 10.1016/S0140-6736(12)62167-9 23395245 PMC4098658

[58] Joseph B Pandit V Sadoun M Zangbar B Fain MJ Friese RS Frailty in surgery. J Trauma Acute Care Surg. (2014) 76:1151–6. 10.1097/TA.0000000000000103 24662884

[59] Robinson TN Eiseman B Wallace JI Church SD McFann KK Pfister SM Redefining geriatric preoperative assessment using frailty, disability and co-morbidity. Ann Surg. (2009) 250:449–55. 10.1097/SLA.0b013e3181b45598 19730176

