# Supplementary material for: Prioritizing patients for hip fracture surgery: the role of frailty and cardiac risk
Source: Front Surg. 2024 Mar 8;11:1367457. doi: 10.3389/fsurg.2024.1367457 (PMC10957751; doi:10.3389/fsurg.2024.1367457)
Supplement: Supplementary file 1 [file Table1.docx]

| **Supplemental Table S1.** Demographic and clinical characteristics of hip fracture patients with OFS 0, after inverse probability weighting | | | |
| --- | --- | --- | --- |
|  | **Surgery ≤24 hours (N = 118,611)** | **Surgery >24 hours (N = 118,546)** | **ASD** |
| Age, median [IQR] | 77 [71.0-81.0] | 77 [71.0-81.0] | 0.002 |
| Sex, n (%) |  |  | <0.001 |
| Female | 82,377 (69.5) | 82,335 (69.5) |  |
| Male | 36,234 (30.5) | 36,210 (30.5) |  |
| Race, n (%) |  |  |  |
| White | 107,549 (90.7) | 107,490 (90.7) | <0.001 |
| Black | 4,186 (3.5) | 4,187 (3.5) | <0.001 |
| Asian | 1,522 (1.3) | 1,520 (1.3) | <0.001 |
| American Indian | 712 (0.6) | 713 (0.6) | <0.001 |
| Pacific Islander | 123 (0.1) | 122 (0.1) | <0.001 |
| Other | 3,934 (3.3) | 3,934 (3.3) | <0.001 |
| Head AIS, n (%) |  |  | <0.001 |
| Injury not present | 115,705 (97.5) | 115,634 (97.5) |  |
| 1 | 2,906 (2.5) | 2,911 (2.5) |  |
| Face AIS, n (%) |  |  | <0.001 |
| Injury not present | 116,145 (97.9) | 116,077 (97.9) |  |
| 1 | 2,467 (2.1) | 2,469 (2.1) |  |
| Neck AIS, n (%) |  |  | <0.001 |
| Injury not present | 118,561 (100.0) | 118,494 (100.0) |  |
| 1 | 50 (0.0) | 52 (0.0) |  |
| Spine AIS, n (%) |  |  | 0.001 |
| Injury not present | 118,500 (99.9) | 118,432 (99.9) |  |
| 1 | 111 (0.1) | 113 (0.1) |  |
| Thorax AIS, n (%) |  |  | 0.001 |
| Injury not present | 117,994 (99.5) | 117,934 (99.5) |  |
| 1 | 617 (0.5) | 612 (0.5) |  |
| Abdomen AIS, n (%) |  |  | <0.001 |
| Injury not present | 118,358 (99.8) | 118,294 (99.8) |  |
| 1 | 253 (0.2) | 251 (0.2) |  |
| Upper extremity AIS, n (%) |  |  | <0.001 |
| Injury not present | 110,754 (93.4) | 110,688 (93.4) |  |
| 1 | 7,857 (6.6) | 7,857 (6.6) |  |
| Lower extremity AIS, n (%) |  |  | 0.005 |
| 3 | 118,600 (100.0) | 118,535 (100.0) |  |
| 4 | 10 (0.0) | 11 (0.0) |  |
| 5 | 2 (0.0) | 0 (0.0) |  |
| External AIS, n (%) |  |  | 0.001 |
| Injury not present | 117,936 (99.4) | 117,867 (99.4) |  |
| 1 | 675 (0.6) | 679 (0.6) |  |
| Type of fracture, n (%) |  |  | <0.001 |
| Cervical | 49,120 (41.4) | 49,096 (41.4) |  |
| Basicervical | 6,984 (5.9) | 6,983 (5.9) |  |
| Pertrochanteric | 58,172 (49.0) | 58,141 (49.0) |  |
| Subtrochanteric | 4,336 (3.7) | 4,326 (3.6) |  |
| Type of surgery, n (%) |  |  | 0.001 |
| Internal fixation | 76,158 (64.2) | 76,076 (64.2) |  |
| Arthroplasty | 42,454 (35.8) | 42,469 (35.8) |  |
| Hypertension, n (%) | 75,071 (63.3) | 75,090 (63.3) | 0.001 |
| Previous myocardial  infarction, n (%) | 1,795 (1.5) | 1,794 (1.5) | <0.001 |
| Congestive heart failure, n (%) | 0 (0.0) | 0 (0.0) | <0.001 |
| Peripheral vascular disease, n (%) | 2,009 (1.7) | 2,005 (1.7) | <0.001 |
| Cerebrovascular disease, n (%) | 5,622 (4.7) | 5,642 (4.8) | 0.001 |
| Dementia, n (%) | 10,818 (9.1) | 10,868 (9.2) | 0.002 |
| Institutionalized, n (%) | 0 (0.0) | 0 (0.0) | <0.001 |
| Non-independent  functional status, n (%) | 0 (0.0) | 0 (0.0) | <0.001 |
| COPD, n (%) | 17,848 (15.0) | 17,822 (15.0) | <0.001 |
| Current smoker, n (%) | 16,234 (13.7) | 16,209 (13.7) | <0.001 |
| Chronic renal failure, n (%) | 3,548 (3.0) | 3,549 (3.0) | <0.001 |
| Diabetes mellitus, n (%) | 27,750 (23.4) | 27,770 (23.4) | 0.001 |
| Cirrhosis, n (%) | 1,178 (1.0) | 1,173 (1.0) | <0.001 |
| Coagulopathy, n (%) | 5,306 (4.5) | 5,293 (4.5) | <0.001 |
| Currently receiving  chemotherapy for cancer, n (%) | 0 (0.0) | 0 (0.0) | <0.001 |
| Metastatic cancer, n (%) | 0 (0.0) | 0 (0.0) | <0.001 |
| Drug use disorder, n (%) | 1,305 (1.1) | 1,299 (1.1) | <0.001 |
| Alcohol use disorder, n (%) | 3,869 (3.3) | 3,874 (3.3) | <0.001 |
| Major psychiatric illness, n (%) | 12,513 (10.5) | 12,523 (10.6) | <0.001 |
| Advanced directive  limiting care, n (%) | 4,906 (4.1) | 4,906 (4.1) | <0.001 |
| An ASD <0.1 is considered balanced.  *OFS, Orthopedic Frailty Score; ASD, Absolute Standardized Difference; AIS, Abbreviated Injury Scale; COPD, Chronic Obstructive Pulmonary Disease* | | | |

| **Supplemental Table S2.** Demographic and clinical characteristics of hip fracture patients with OFS 1, after inverse probability weighting | | | |
| --- | --- | --- | --- |
|  | **Surgery ≤24 hours (N = 87,251)** | **Surgery >24 hours (N = 87,213)** | **ASD** |
| Age, median [IQR] | 83 [77.0-87.0] | 83 [77.0-87.0] | 0.001 |
| Sex, n (%) |  |  | <0.001 |
| Female | 59,323 (68.0) | 59,311 (68.0) |  |
| Male | 27,928 (32.0) | 27,902 (32.0) |  |
| Race, n (%) |  |  |  |
| White | 79,004 (90.5) | 78,979 (90.6) | <0.001 |
| Black | 3,488 (4.0) | 3,485 (4.0) | <0.001 |
| Asian | 1,000 (1.1) | 999 (1.1) | <0.001 |
| American Indian | 508 (0.6) | 504 (0.6) | 0.001 |
| Pacific Islander | 90 (0.1) | 91 (0.1) | <0.001 |
| Other | 2,765 (3.2) | 2,760 (3.2) | <0.001 |
| Head AIS, n (%) |  |  | <0.001 |
| Injury not present | 84,292 (96.6) | 84,261 (96.6) |  |
| 1 | 2,959 (3.4) | 2,951 (3.4) |  |
| Face AIS, n (%) |  |  | <0.001 |
| Injury not present | 84,853 (97.3) | 84,818 (97.3) |  |
| 1 | 2,398 (2.7) | 2,395 (2.7) |  |
| Neck AIS, n (%) |  |  | 0.001 |
| Injury not present | 87,176 (99.9) | 87,137 (99.9) |  |
| 1 | 75 (0.1) | 76 (0.1) |  |
| Spine AIS, n (%) |  |  | <0.001 |
| Injury not present | 87,163 (99.9) | 87,126 (99.9) |  |
| 1 | 87 (0.1) | 87 (0.1) |  |
| Thorax AIS, n (%) |  |  | 0.001 |
| Injury not present | 86,682 (99.3) | 86,640 (99.3) |  |
| 1 | 569 (0.7) | 573 (0.7) |  |
| Abdomen AIS, n (%) |  |  | <0.001 |
| Injury not present | 86,989 (99.7) | 86,952 (99.7) |  |
| 1 | 261 (0.3) | 261 (0.3) |  |
| Upper extremity AIS, n (%) |  |  | <0.001 |
| Injury not present | 80,261 (92.0) | 80,231 (92.0) |  |
| 1 | 6,990 (8.0) | 6,981 (8.0) |  |
| Lower extremity AIS, n (%) |  |  | 0.004 |
| 3 | 87,239 (100.0) | 87,201 (100.0) |  |
| 4 | 12 (0.0) | 11 (0.0) |  |
| 5 | 0 (0.0) | 1 (0.0) |  |
| External AIS, n (%) |  |  | <0.001 |
| Injury not present | 86,640 (99.3) | 86,605 (99.3) |  |
| 1 | 611 (0.7) | 608 (0.7) |  |
| Type of fracture, n (%) |  |  | <0.001 |
| Cervical | 34,659 (39.7) | 34,648 (39.7) |  |
| Basicervical | 4,960 (5.7) | 4,965 (5.7) |  |
| Pertrochanteric | 44,699 (51.2) | 44,670 (51.2) |  |
| Subtrochanteric | 2,932 (3.4) | 2,929 (3.4) |  |
| Type of surgery, n (%) |  |  | <0.001 |
| Internal fixation | 56,844 (65.2) | 56,830 (65.2) |  |
| Arthroplasty | 30,406 (34.8) | 30,383 (34.8) |  |
| Hypertension, n (%) | 61,021 (69.9) | 61,003 (69.9) | <0.001 |
| Previous myocardial  infarction, n (%) | 1,693 (1.9) | 1,689 (1.9) | <0.001 |
| Congestive heart failure, n (%) | 10,824 (12.4) | 10,800 (12.4) | 0.001 |
| Peripheral vascular disease, n (%) | 2,382 (2.7) | 2,380 (2.7) | <0.001 |
| Cerebrovascular disease, n (%) | 6,411 (7.3) | 6,406 (7.3) | <0.001 |
| Dementia, n (%) | 17,599 (20.2) | 17,609 (20.2) | <0.001 |
| Institutionalized, n (%) | 8,198 (9.4) | 8,206 (9.4) | <0.001 |
| Non-independent  functional status, n (%) | 26,761 (30.7) | 26,743 (30.7) | <0.001 |
| COPD, n (%) | 14,758 (16.9) | 14,739 (16.9) | <0.001 |
| Current smoker, n (%) | 7,576 (8.7) | 7,564 (8.7) | <0.001 |
| Chronic renal failure, n (%) | 4,074 (4.7) | 4,067 (4.7) | <0.001 |
| Diabetes mellitus, n (%) | 21,460 (24.6) | 21,448 (24.6) | <0.001 |
| Cirrhosis, n (%) | 843 (1.0) | 840 (1.0) | <0.001 |
| Coagulopathy, n (%) | 4,948 (5.7) | 4,919 (5.6) | 0.001 |
| Currently receiving  chemotherapy for cancer, n (%) | 1,501 (1.7) | 1,501 (1.7) | <0.001 |
| Metastatic cancer, n (%) | 1,767 (2.0) | 1,769 (2.0) | <0.001 |
| Drug use disorder, n (%) | 742 (0.8) | 741 (0.8) | <0.001 |
| Alcohol use disorder, n (%) | 1,620 (1.9) | 1,618 (1.9) | <0.001 |
| Major psychiatric illness, n (%) | 10,726 (12.3) | 10,727 (12.3) | <0.001 |
| Advanced directive  limiting care, n (%) | 8,126 (9.3) | 8,115 (9.3) | <0.001 |
| An ASD <0.1 is considered balanced.  *OFS, Orthopedic Frailty Score; ASD, Absolute Standardized Difference; AIS, Abbreviated Injury Scale; COPD, Chronic Obstructive Pulmonary Disease* | | | |

| **Supplemental Table S3.** Demographic and clinical characteristics of hip fracture patients with OFS 2, after inverse probability weighting | | | |
| --- | --- | --- | --- |
|  | **Surgery ≤24 hours (N = 37,800)** | **Surgery >24 hours (N = 37,787)** | **ASD** |
| Age, median [IQR] | 86 [81.0-88.0] | 86 [81.0-87.0] | 0.001 |
| Sex, n (%) |  |  | <0.001 |
| Female | 25,605 (67.7) | 25,597 (67.7) |  |
| Male | 12,195 (32.3) | 12,190 (32.3) |  |
| Race, n (%) |  |  |  |
| White | 34,428 (91.1) | 34,421 (91.1) | <0.001 |
| Black | 1,579 (4.2) | 1,578 (4.2) | <0.001 |
| Asian | 419 (1.1) | 418 (1.1) | <0.001 |
| American Indian | 173 (0.5) | 179 (0.5) | 0.002 |
| Pacific Islander | 30 (0.1) | 29 (0.1) | 0.001 |
| Other | 976 (2.6) | 968 (2.6) | 0.001 |
| Head AIS, n (%) |  |  | <0.001 |
| Injury not present | 36,243 (95.9) | 36,230 (95.9) |  |
| 1 | 1,557 (4.1) | 1,557 (4.1) |  |
| Face AIS, n (%) |  |  | <0.001 |
| Injury not present | 36,528 (96.6) | 36,515 (96.6) |  |
| 1 | 1,272 (3.4) | 1,272 (3.4) |  |
| Neck AIS, n (%) |  |  | <0.001 |
| Injury not present | 37,776 (99.9) | 37,763 (99.9) |  |
| 1 | 24 (0.1) | 24 (0.1) |  |
| Spine AIS, n (%) |  |  | 0.001 |
| Injury not present | 37,753 (99.9) | 37,739 (99.9) |  |
| 1 | 47 (0.1) | 48 (0.1) |  |
| Thorax AIS, n (%) |  |  | <0.001 |
| Injury not present | 37,527 (99.3) | 37,513 (99.3) |  |
| 1 | 273 (0.7) | 274 (0.7) |  |
| Abdomen AIS, n (%) |  |  | <0.001 |
| Injury not present | 37,664 (99.6) | 37,650 (99.6) |  |
| 1 | 136 (0.4) | 137 (0.4) |  |
| Upper extremity AIS, n (%) |  |  | <0.001 |
| Injury not present | 34,370 (90.9) | 34,362 (90.9) |  |
| 1 | 3,430 (9.1) | 3,425 (9.1) |  |
| Lower extremity AIS, n (%) |  |  | <0.001 |
| 3 | 37,797 (100.0) | 37,784 (100.0) |  |
| 4 | 3 (0.0) | 3 (0.0) |  |
| 5 | 0 (0.0) | 0 (0.0) |  |
| External AIS, n (%) |  |  | <0.001 |
| Injury not present | 37,493 (99.2) | 37,479 (99.2) |  |
| 1 | 307 (0.8) | 308 (0.8) |  |
| Type of fracture, n (%) |  |  | 0.001 |
| Cervical | 14,955 (39.6) | 14,948 (39.6) |  |
| Basicervical | 2,149 (5.7) | 2,152 (5.7) |  |
| Pertrochanteric | 19,566 (51.8) | 19,559 (51.8) |  |
| Subtrochanteric | 1,130 (3.0) | 1,128 (3.0) |  |
| Type of surgery, n (%) |  |  | <0.001 |
| Internal fixation | 24,774 (65.5) | 24,771 (65.6) |  |
| Arthroplasty | 13,026 (34.5) | 13,016 (34.4) |  |
| Hypertension, n (%) | 27,565 (72.9) | 27,546 (72.9) | 0.001 |
| Previous myocardial  infarction, n (%) | 754 (2.0) | 752 (2.0) | <0.001 |
| Congestive heart failure, n (%) | 10,452 (27.7) | 10,440 (27.6) | 0.001 |
| Peripheral vascular disease, n (%) | 1,331 (3.5) | 1,326 (3.5) | 0.001 |
| Cerebrovascular disease, n (%) | 3,245 (8.6) | 3,240 (8.6) | <0.001 |
| Dementia, n (%) | 13,678 (36.2) | 13,686 (36.2) | 0.001 |
| Institutionalized, n (%) | 13,759 (36.4) | 13,762 (36.4) | <0.001 |
| Non-independent  functional status, n (%) | 25,860 (68.4) | 25,845 (68.4) | <0.001 |
| COPD, n (%) | 7,181 (19.0) | 7,174 (19.0) | <0.001 |
| Current smoker, n (%) | 2,247 (5.9) | 2,250 (6.0) | <0.001 |
| Chronic renal failure, n (%) | 1,982 (5.2) | 1,978 (5.2) | <0.001 |
| Diabetes mellitus, n (%) | 9,438 (25.0) | 9,431 (25.0) | <0.001 |
| Cirrhosis, n (%) | 348 (0.9) | 347 (0.9) | <0.001 |
| Coagulopathy, n (%) | 2,255 (6.0) | 2,244 (5.9) | 0.001 |
| Currently receiving  chemotherapy for cancer, n (%) | 880 (2.3) | 882 (2.3) | 0.001 |
| Metastatic cancer, n (%) | 1,216 (3.2) | 1,213 (3.2) | <0.001 |
| Drug use disorder, n (%) | 284 (0.8) | 282 (0.7) | <0.001 |
| Alcohol use disorder, n (%) | 471 (1.2) | 474 (1.3) | 0.001 |
| Major psychiatric illness, n (%) | 5,865 (15.5) | 5,863 (15.5) | <0.001 |
| Advanced directive  limiting care, n (%) | 6,851 (18.1) | 6,854 (18.1) | <0.001 |
| An ASD <0.1 is considered balanced.  *OFS, Orthopedic Frailty Score; ASD, Absolute Standardized Difference; AIS, Abbreviated Injury Scale; COPD, Chronic Obstructive Pulmonary Disease* | | | |

| **Supplemental Table S4.** Demographic and clinical characteristics of hip fracture patients with OFS 3, after inverse probability weighting | | | |
| --- | --- | --- | --- |
|  | **Surgery ≤24 hours (N = 9,834)** | **Surgery >24 hours (N = 9,835)** | **ASD** |
| Age, median [IQR] | 87 [85.0-88.0] | 87 [85.0-88.0] | 0.001 |
| Sex, n (%) |  |  | <0.001 |
| Female | 6,705 (68.2) | 6,707 (68.2) |  |
| Male | 3,129 (31.8) | 3,128 (31.8) |  |
| Race, n (%) |  |  |  |
| White | 9,057 (92.1) | 9,055 (92.1) | 0.001 |
| Black | 395 (4.0) | 397 (4.0) | 0.001 |
| Asian | 87 (0.9) | 87 (0.9) | <0.001 |
| American Indian | 42 (0.4) | 41 (0.4) | 0.001 |
| Pacific Islander | 9 (0.1) | 10 (0.1) | 0.001 |
| Other | 199 (2.0) | 201 (2.0) | 0.002 |
| Head AIS, n (%) |  |  | 0.003 |
| Injury not present | 9,324 (94.8) | 9,331 (94.9) |  |
| 1 | 510 (5.2) | 504 (5.1) |  |
| Face AIS, n (%) |  |  | <0.001 |
| Injury not present | 9,467 (96.3) | 9,467 (96.3) |  |
| 1 | 367 (3.7) | 367 (3.7) |  |
| Neck AIS, n (%) |  |  | 0.001 |
| Injury not present | 9,826 (99.9) | 9,827 (99.9) |  |
| 1 | 8 (0.1) | 8 (0.1) |  |
| Spine AIS, n (%) |  |  | 0.005 |
| Injury not present | 9,822 (99.9) | 9,825 (99.9) |  |
| 1 | 12 (0.1) | 10 (0.1) |  |
| Thorax AIS, n (%) |  |  | 0.003 |
| Injury not present | 9,751 (99.2) | 9,754 (99.2) |  |
| 1 | 83 (0.8) | 80 (0.8) |  |
| Abdomen AIS, n (%) |  |  | 0.004 |
| Injury not present | 9,790 (99.6) | 9,794 (99.6) |  |
| 1 | 44 (0.4) | 41 (0.4) |  |
| Upper extremity AIS, n (%) |  |  | 0.001 |
| Injury not present | 8,804 (89.5) | 8,807 (89.6) |  |
| 1 | 1,030 (10.5) | 1,028 (10.4) |  |
| Lower extremity AIS, n (%) |  |  | 0.007 |
| 3 | 9,831 (100.0) | 9,830 (99.9) |  |
| 4 | 4 (0.0) | 5 (0.1) |  |
| 5 | 0 (0.0) | 0 (0.0) |  |
| External AIS, n (%) |  |  | <0.001 |
| Injury not present | 9,755 (99.2) | 9,756 (99.2) |  |
| 1 | 79 (0.8) | 79 (0.8) |  |
| Type of fracture, n (%) |  |  | 0.001 |
| Cervical | 3,696 (37.6) | 3,692 (37.5) |  |
| Basicervical | 557 (5.7) | 558 (5.7) |  |
| Pertrochanteric | 5,282 (53.7) | 5,286 (53.8) |  |
| Subtrochanteric | 299 (3.0) | 298 (3.0) |  |
| Type of surgery, n (%) |  |  | <0.001 |
| Internal fixation | 6,606 (67.2) | 6,608 (67.2) |  |
| Arthroplasty | 3,228 (32.8) | 3,227 (32.8) |  |
| Hypertension, n (%) | 7,352 (74.8) | 7,356 (74.8) | 0.001 |
| Previous myocardial  infarction, n (%) | 188 (1.9) | 188 (1.9) | <0.001 |
| Congestive heart failure, n (%) | 4,541 (46.2) | 4,538 (46.1) | 0.001 |
| Peripheral vascular disease, n (%) | 399 (4.1) | 397 (4.0) | 0.001 |
| Cerebrovascular disease, n (%) | 872 (8.9) | 875 (8.9) | 0.001 |
| Dementia, n (%) | 4,870 (49.5) | 4,872 (49.5) | <0.001 |
| Institutionalized, n (%) | 6,924 (70.4) | 6,929 (70.5) | 0.001 |
| Non-independent  functional status, n (%) | 8,934 (90.8) | 8,935 (90.9) | <0.001 |
| COPD, n (%) | 2,033 (20.7) | 2,033 (20.7) | <0.001 |
| Current smoker, n (%) | 386 (3.9) | 385 (3.9) | <0.001 |
| Chronic renal failure, n (%) | 581 (5.9) | 577 (5.9) | 0.002 |
| Diabetes mellitus, n (%) | 2,374 (24.1) | 2,375 (24.1) | <0.001 |
| Cirrhosis, n (%) | 75 (0.8) | 73 (0.7) | 0.002 |
| Coagulopathy, n (%) | 643 (6.5) | 636 (6.5) | 0.003 |
| Currently receiving  chemotherapy for cancer, n (%) | 281 (2.9) | 276 (2.8) | 0.003 |
| Metastatic cancer, n (%) | 464 (4.7) | 466 (4.7) | 0.001 |
| Drug use disorder, n (%) | 43 (0.4) | 43 (0.4) | 0.001 |
| Alcohol use disorder, n (%) | 80 (0.8) | 80 (0.8) | <0.001 |
| Major psychiatric illness, n (%) | 1,608 (16.3) | 1,609 (16.4) | <0.001 |
| Advanced directive  limiting care, n (%) | 2,833 (28.8) | 2,837 (28.8) | 0.001 |
| An ASD <0.1 is considered balanced.  *OFS, Orthopedic Frailty Score; ASD, Absolute Standardized Difference; AIS, Abbreviated Injury Scale; COPD, Chronic Obstructive Pulmonary Disease* | | | |

| **Supplemental Table S5.** Demographic and clinical characteristics of hip fracture patients with OFS ≥4, after inverse probability weighting | | | |
| --- | --- | --- | --- |
|  | **Surgery ≤24 hours (N = 952)** | **Surgery >24 hours (N = 957)** | **ASD** |
| Age, median [IQR] | 87 [86.0-88.0] | 87 [86.0-88.0] | 0.009 |
| Sex, n (%) |  |  | 0.009 |
| Female | 637 (66.9) | 644 (67.4) |  |
| Male | 315 (33.1) | 312 (32.6) |  |
| Race, n (%) |  |  |  |
| White | 898 (94.4) | 902 (94.3) | 0.005 |
| Black | 21 (2.2) | 22 (2.3) | 0.003 |
| Asian | 4 (0.5) | 4 (0.4) | 0.004 |
| American Indian | 5 (0.5) | 4 (0.4) | 0.019 |
| Pacific Islander | 1 (0.1) | 0 (0.0) | 0.046 |
| Other | 22 (2.3) | 24 (2.6) | 0.014 |
| Head AIS, n (%) |  |  | 0.012 |
| Injury not present | 915 (96.2) | 918 (95.9) |  |
| 1 | 36 (3.8) | 39 (4.1) |  |
| Face AIS, n (%) |  |  | 0.003 |
| Injury not present | 906 (95.2) | 910 (95.1) |  |
| 1 | 46 (4.8) | 46 (4.9) |  |
| Neck AIS, n (%) |  |  | 0.079 |
| Injury not present | 952 (100.0) | 954 (99.7) |  |
| 1 | 0 (0.0) | 3 (0.3) |  |
| Spine AIS, n (%) |  |  | 0.046 |
| Injury not present | 952 (100.0) | 956 (99.9) |  |
| 1 | 0 (0.0) | 1 (0.1) |  |
| Thorax AIS, n (%) |  |  | 0.004 |
| Injury not present | 942 (99.0) | 947 (98.9) |  |
| 1 | 10 (1.0) | 10 (1.1) |  |
| Abdomen AIS, n (%) |  |  | 0.002 |
| Injury not present | 947 (99.5) | 952 (99.5) |  |
| 1 | 5 (0.5) | 5 (0.5) |  |
| Upper extremity AIS, n (%) |  |  | 0.008 |
| Injury not present | 863 (90.7) | 866 (90.5) |  |
| 1 | 88 (9.3) | 91 (9.5) |  |
| Lower extremity AIS, n (%) |  |  | <0.001 |
| 3 | 952 (100.0) | 957 (100.0) |  |
| 4 | 0 (0.0) | 0 (0.0) |  |
| 5 | 0 (0.0) | 0 (0.0) |  |
| External AIS, n (%) |  |  | 0.005 |
| Injury not present | 940 (98.8) | 944 (98.7) |  |
| 1 | 12 (1.2) | 12 (1.3) |  |
| Type of fracture, n (%) |  |  | 0.006 |
| Cervical | 363 (38.2) | 363 (37.9) |  |
| Basicervical | 57 (6.0) | 58 (6.0) |  |
| Pertrochanteric | 493 (51.8) | 498 (52.0) |  |
| Subtrochanteric | 39 (4.1) | 38 (4.0) |  |
| Type of surgery, n (%) |  |  | 0.003 |
| Internal fixation | 646 (67.9) | 649 (67.8) |  |
| Arthroplasty | 305 (32.1) | 308 (32.2) |  |
| Hypertension, n (%) | 743 (78.1) | 746 (78.0) | 0.002 |
| Previous myocardial  infarction, n (%) | 36 (3.8) | 36 (3.7) | 0.003 |
| Congestive heart failure, n (%) | 901 (94.7) | 907 (94.7) | 0.001 |
| Peripheral vascular disease, n (%) | 44 (4.6) | 45 (4.7) | 0.001 |
| Cerebrovascular disease, n (%) | 94 (9.9) | 96 (10.0) | 0.002 |
| Dementia, n (%) | 529 (55.6) | 533 (55.7) | 0.001 |
| Institutionalized, n (%) | 909 (95.5) | 916 (95.7) | 0.008 |
| Non-independent  functional status, n (%) | 933 (98.0) | 940 (98.2) | 0.015 |
| COPD, n (%) | 274 (28.8) | 275 (28.8) | <0.001 |
| Current smoker, n (%) | 20 (2.1) | 20 (2.1) | 0.004 |
| Chronic renal failure, n (%) | 60 (6.3) | 62 (6.5) | 0.007 |
| Diabetes mellitus, n (%) | 226 (23.8) | 230 (24.0) | 0.006 |
| Cirrhosis, n (%) | 4 (0.5) | 5 (0.5) | 0.005 |
| Coagulopathy, n (%) | 77 (8.1) | 78 (8.2) | 0.003 |
| Currently receiving  chemotherapy for cancer, n (%) | 44 (4.6) | 42 (4.4) | 0.011 |
| Metastatic cancer, n (%) | 104 (11.0) | 100 (10.5) | 0.016 |
| Drug use disorder, n (%) | 2 (0.2) | 2 (0.2) | <0.001 |
| Alcohol use disorder, n (%) | 8 (0.9) | 9 (0.9) | 0.001 |
| Major psychiatric illness, n (%) | 200 (21.0) | 196 (20.5) | 0.013 |
| Advanced directive  limiting care, n (%) | 373 (39.2) | 371 (38.7) | 0.010 |
| An ASD <0.1 is considered balanced.  *OFS, Orthopedic Frailty Score; ASD, Absolute Standardized Difference; AIS, Abbreviated Injury Scale; COPD, Chronic Obstructive Pulmonary Disease* | | | |

| **Supplemental Table S6.** Demographic and clinical characteristics of hip fracture patients with RCRI 0, after inverse probability weighting | | | |
| --- | --- | --- | --- |
|  | **Surgery ≤24 hours (N = 159,161)** | **Surgery >24 hours (N = 159,087)** | **ASD** |
| Age, median [IQR] | 80 [74.0-85.0] | 81 [74.0-85.0] | 0.002 |
| Sex, n (%) |  |  | <0.001 |
| Female | 115,115 (72.3) | 115,053 (72.3) |  |
| Male | 44,046 (27.7) | 44,034 (27.7) |  |
| Race, n (%) |  |  |  |
| White | 146,965 (92.3) | 146,889 (92.3) | <0.001 |
| Black | 4,581 (2.9) | 4,585 (2.9) | <0.001 |
| Asian | 1,697 (1.1) | 1,695 (1.1) | <0.001 |
| American Indian | 747 (0.5) | 751 (0.5) | <0.001 |
| Pacific Islander | 127 (0.1) | 128 (0.1) | <0.001 |
| Other | 4,252 (2.7) | 4,250 (2.7) | <0.001 |
| Head AIS, n (%) |  |  | <0.001 |
| Injury not present | 154,474 (97.1) | 154,399 (97.1) |  |
| 1 | 4,687 (2.9) | 4,688 (2.9) |  |
| Face AIS, n (%) |  |  | <0.001 |
| Injury not present | 155,143 (97.5) | 155,068 (97.5) |  |
| 1 | 4,018 (2.5) | 4,019 (2.5) |  |
| Neck AIS, n (%) |  |  | <0.001 |
| Injury not present | 159,066 (99.9) | 158,990 (99.9) |  |
| 1 | 95 (0.1) | 97 (0.1) |  |
| Spine AIS, n (%) |  |  | <0.001 |
| Injury not present | 159,012 (99.9) | 158,936 (99.9) |  |
| 1 | 149 (0.1) | 151 (0.1) |  |
| Thorax AIS, n (%) |  |  | <0.001 |
| Injury not present | 158,263 (99.4) | 158,189 (99.4) |  |
| 1 | 898 (0.6) | 898 (0.6) |  |
| Abdomen AIS, n (%) |  |  | <0.001 |
| Injury not present | 158,834 (99.8) | 158,760 (99.8) |  |
| 1 | 327 (0.2) | 327 (0.2) |  |
| Upper extremity AIS, n (%) |  |  | <0.001 |
| Injury not present | 147,358 (92.6) | 147,275 (92.6) |  |
| 1 | 11,803 (7.4) | 11,812 (7.4) |  |
| Lower extremity AIS, n (%) |  |  | 0.004 |
| 3 | 159,143 (100.0) | 159,071 (100.0) |  |
| 4 | 17 (0.0) | 16 (0.0) |  |
| 5 | 2 (0.0) | 0 (0.0) |  |
| External AIS, n (%) |  |  | <0.001 |
| Injury not present | 158,173 (99.4) | 158,097 (99.4) |  |
| 1 | 988 (0.6) | 990 (0.6) |  |
| Type of fracture, n (%) |  |  | 0.001 |
| Cervical | 66,192 (41.6) | 66,189 (41.6) |  |
| Basicervical | 9,482 (6.0) | 9,496 (6.0) |  |
| Pertrochanteric | 78,152 (49.1) | 78,080 (49.1) |  |
| Subtrochanteric | 5,336 (3.4) | 5,322 (3.3) |  |
| Type of surgery, n (%) |  |  | 0.001 |
| Internal fixation | 101,494 (63.8) | 101,395 (63.7) |  |
| Arthroplasty | 57,667 (36.2) | 57,692 (36.3) |  |
| Hypertension, n (%) | 96,198 (60.4) | 96,198 (60.5) | 0.001 |
| Previous myocardial  infarction, n (%) | 0 (0.0) | 0 (0.0) | <0.001 |
| Congestive heart failure, n (%) | 0 (0.0) | 0 (0.0) | <0.001 |
| Peripheral vascular disease, n (%) | 2,499 (1.6) | 2,496 (1.6) | <0.001 |
| Cerebrovascular disease, n (%) | 0 (0.0) | 0 (0.0) | <0.001 |
| Dementia, n (%) | 30,409 (19.1) | 30,493 (19.2) | 0.002 |
| Institutionalized, n (%) | 17,578 (11.0) | 17,624 (11.1) | 0.001 |
| Non-independent  functional status, n (%) | 34,784 (21.9) | 34,828 (21.9) | 0.001 |
| COPD, n (%) | 23,308 (14.6) | 23,290 (14.6) | <0.001 |
| Current smoker, n (%) | 17,470 (11.0) | 17,440 (11.0) | <0.001 |
| Chronic renal failure, n (%) | 0 (0.0) | 0 (0.0) | <0.001 |
| Diabetes mellitus, n (%) | 0 (0.0) | 0 (0.0) | <0.001 |
| Cirrhosis, n (%) | 1,003 (0.6) | 1,005 (0.6) | <0.001 |
| Coagulopathy, n (%) | 6,528 (4.1) | 6,518 (4.1) | <0.001 |
| Currently receiving  chemotherapy for cancer, n (%) | 1,751 (1.1) | 1,756 (1.1) | <0.001 |
| Metastatic cancer, n (%) | 2,208 (1.4) | 2,210 (1.4) | <0.001 |
| Drug use disorder, n (%) | 1,506 (0.9) | 1,502 (0.9) | <0.001 |
| Alcohol use disorder, n (%) | 4,376 (2.7) | 4,376 (2.8) | <0.001 |
| Major psychiatric illness, n (%) | 18,760 (11.8) | 18,770 (11.8) | <0.001 |
| Advanced directive  limiting care, n (%) | 13,459 (8.5) | 13,482 (8.5) | 0.001 |
| An ASD <0.1 is considered balanced.  *RCRI, Revised Cardiac Risk Index; ASD, Absolute Standardized Difference; AIS, Abbreviated Injury Scale; COPD, Chronic Obstructive Pulmonary Disease* | | | |

| **Supplemental Table S7.** Demographic and clinical characteristics of hip fracture patients with RCRI 1, after inverse probability weighting | | | |
| --- | --- | --- | --- |
|  | **Surgery ≤24 hours (N = 72,545)** | **Surgery >24 hours (N = 72,531)** | **ASD** |
| Age, median [IQR] | 80 [74.0-85.0] | 80 [74.0-85.0] | <0.001 |
| Sex, n (%) |  |  | 0.001 |
| Female | 46,954 (64.7) | 46,967 (64.8) |  |
| Male | 25,591 (35.3) | 25,564 (35.2) |  |
| Race, n (%) |  |  |  |
| White | 64,473 (88.9) | 64,472 (88.9) | <0.001 |
| Black | 3,372 (4.6) | 3,370 (4.6) | <0.001 |
| Asian | 1,028 (1.4) | 1,027 (1.4) | <0.001 |
| American Indian | 542 (0.7) | 544 (0.7) | <0.001 |
| Pacific Islander | 82 (0.1) | 81 (0.1) | <0.001 |
| Other | 2,706 (3.7) | 2,700 (3.7) | <0.001 |
| Head AIS, n (%) |  |  | <0.001 |
| Injury not present | 70,091 (96.6) | 70,081 (96.6) |  |
| 1 | 2,454 (3.4) | 2,451 (3.4) |  |
| Face AIS, n (%) |  |  | <0.001 |
| Injury not present | 70,681 (97.4) | 70,669 (97.4) |  |
| 1 | 1,864 (2.6) | 1,862 (2.6) |  |
| Neck AIS, n (%) |  |  | 0.001 |
| Injury not present | 72,502 (99.9) | 72,490 (99.9) |  |
| 1 | 43 (0.1) | 42 (0.1) |  |
| Spine AIS, n (%) |  |  | 0.001 |
| Injury not present | 72,458 (99.9) | 72,447 (99.9) |  |
| 1 | 87 (0.1) | 84 (0.1) |  |
| Thorax AIS, n (%) |  |  | <0.001 |
| Injury not present | 72,101 (99.4) | 72,088 (99.4) |  |
| 1 | 444 (0.6) | 444 (0.6) |  |
| Abdomen AIS, n (%) |  |  | <0.001 |
| Injury not present | 72,292 (99.7) | 72,278 (99.7) |  |
| 1 | 253 (0.3) | 253 (0.3) |  |
| Upper extremity AIS, n (%) |  |  | <0.001 |
| Injury not present | 66,875 (92.2) | 66,866 (92.2) |  |
| 1 | 5,670 (7.8) | 5,666 (7.8) |  |
| Lower extremity AIS, n (%) |  |  | 0.001 |
| 3 | 72,535 (100.0) | 72,522 (100.0) |  |
| 4 | 10 (0.0) | 9 (0.0) |  |
| 5 | 0 (0.0) | 0 (0.0) |  |
| External AIS, n (%) |  |  | <0.001 |
| Injury not present | 72,036 (99.3) | 72,024 (99.3) |  |
| 1 | 509 (0.7) | 508 (0.7) |  |
| Type of fracture, n (%) |  |  | 0.001 |
| Cervical | 28,063 (38.7) | 28,043 (38.7) |  |
| Basicervical | 4,061 (5.6) | 4,064 (5.6) |  |
| Pertrochanteric | 37,883 (52.2) | 37,884 (52.2) |  |
| Subtrochanteric | 2,538 (3.5) | 2,541 (3.5) |  |
| Type of surgery, n (%) |  |  | <0.001 |
| Internal fixation | 48,088 (66.3) | 48,094 (66.3) |  |
| Arthroplasty | 24,457 (33.7) | 24,437 (33.7) |  |
| Hypertension, n (%) | 56,873 (78.4) | 56,865 (78.4) | <0.001 |
| Previous myocardial  infarction, n (%) | 1,725 (2.4) | 1,722 (2.4) | <0.001 |
| Congestive heart failure, n (%) | 13,621 (18.8) | 13,607 (18.8) | <0.001 |
| Peripheral vascular disease, n (%) | 2,314 (3.2) | 2,306 (3.2) | 0.001 |
| Cerebrovascular disease, n (%) | 8,826 (12.2) | 8,833 (12.2) | <0.001 |
| Dementia, n (%) | 13,246 (18.3) | 13,272 (18.3) | 0.001 |
| Institutionalized, n (%) | 9,017 (12.4) | 9,030 (12.5) | 0.001 |
| Non-independent  functional status, n (%) | 20,212 (27.9) | 20,211 (27.9) | <0.001 |
| COPD, n (%) | 13,254 (18.3) | 13,247 (18.3) | <0.001 |
| Current smoker, n (%) | 6,957 (9.6) | 6,952 (9.6) | <0.001 |
| Chronic renal failure, n (%) | 3,594 (5.0) | 3,593 (5.0) | <0.001 |
| Diabetes mellitus, n (%) | 43,011 (59.3) | 43,007 (59.3) | <0.001 |
| Cirrhosis, n (%) | 972 (1.3) | 969 (1.3) | <0.001 |
| Coagulopathy, n (%) | 4,584 (6.3) | 4,566 (6.3) | 0.001 |
| Currently receiving  chemotherapy for cancer, n (%) | 765 (1.1) | 763 (1.1) | <0.001 |
| Metastatic cancer, n (%) | 1,013 (1.4) | 1,015 (1.4) | <0.001 |
| Drug use disorder, n (%) | 652 (0.9) | 649 (0.9) | <0.001 |
| Alcohol use disorder, n (%) | 1,317 (1.8) | 1,318 (1.8) | <0.001 |
| Major psychiatric illness, n (%) | 9,111 (12.6) | 9,120 (12.6) | <0.001 |
| Advanced directive  limiting care, n (%) | 6,970 (9.6) | 6,968 (9.6) | <0.001 |
| An ASD <0.1 is considered balanced.  *RCRI, Revised Cardiac Risk Index; ASD, Absolute Standardized Difference; AIS, Abbreviated Injury Scale; COPD, Chronic Obstructive Pulmonary Disease* | | | |

| **Supplemental Table S8.** Demographic and clinical characteristics of hip fracture patients with RCRI 2, after inverse probability weighting | | | |
| --- | --- | --- | --- |
|  | **Surgery ≤24 hours (N = 18,676)** | **Surgery >24 hours (N = 18,679)** | **ASD** |
| Age, median [IQR] | 79 [73.0-84.0] | 79 [73.0-84.0] | 0.001 |
| Sex, n (%) |  |  | <0.001 |
| Female | 10,510 (56.3) | 10,512 (56.3) |  |
| Male | 8,166 (43.7) | 8,168 (43.7) |  |
| Race, n (%) |  |  |  |
| White | 16,099 (86.2) | 16,105 (86.2) | 0.001 |
| Black | 1,328 (7.1) | 1,327 (7.1) | <0.001 |
| Asian | 262 (1.4) | 263 (1.4) | <0.001 |
| American Indian | 126 (0.7) | 126 (0.7) | <0.001 |
| Pacific Islander | 34 (0.2) | 34 (0.2) | <0.001 |
| Other | 746 (4.0) | 743 (4.0) | 0.001 |
| Head AIS, n (%) |  |  | 0.001 |
| Injury not present | 18,013 (96.5) | 18,018 (96.5) |  |
| 1 | 663 (3.5) | 661 (3.5) |  |
| Face AIS, n (%) |  |  | 0.001 |
| Injury not present | 18,153 (97.2) | 18,154 (97.2) |  |
| 1 | 523 (2.8) | 526 (2.8) |  |
| Neck AIS, n (%) |  |  | 0.001 |
| Injury not present | 18,658 (99.9) | 18,660 (99.9) |  |
| 1 | 18 (0.1) | 19 (0.1) |  |
| Spine AIS, n (%) |  |  | <0.001 |
| Injury not present | 18,652 (99.9) | 18,655 (99.9) |  |
| 1 | 24 (0.1) | 24 (0.1) |  |
| Thorax AIS, n (%) |  |  | <0.001 |
| Injury not present | 18,510 (99.1) | 18,513 (99.1) |  |
| 1 | 166 (0.9) | 166 (0.9) |  |
| Abdomen AIS, n (%) |  |  | <0.001 |
| Injury not present | 18,589 (99.5) | 18,593 (99.5) |  |
| 1 | 87 (0.5) | 87 (0.5) |  |
| Upper extremity AIS, n (%) |  |  | <0.001 |
| Injury not present | 17,089 (91.5) | 17,094 (91.5) |  |
| 1 | 1,587 (8.5) | 1,586 (8.5) |  |
| Lower extremity AIS, n (%) |  |  | 0.017 |
| 3 | 18,676 (100.0) | 18,677 (100.0) |  |
| 4 | 0 (0.0) | 2 (0.0) |  |
| 5 | 0 (0.0) | 1 (0.0) |  |
| External AIS, n (%) |  |  | 0.001 |
| Injury not present | 18,535 (99.2) | 18,536 (99.2) |  |
| 1 | 141 (0.8) | 143 (0.8) |  |
| Type of fracture, n (%) |  |  | 0.001 |
| Cervical | 7,034 (37.7) | 7,028 (37.6) |  |
| Basicervical | 953 (5.1) | 956 (5.1) |  |
| Pertrochanteric | 9,995 (53.5) | 10,000 (53.5) |  |
| Subtrochanteric | 694 (3.7) | 695 (3.7) |  |
| Type of surgery, n (%) |  |  | <0.001 |
| Internal fixation | 12,606 (67.5) | 12,609 (67.5) |  |
| Arthroplasty | 6,070 (32.5) | 6,070 (32.5) |  |
| Hypertension, n (%) | 15,310 (82.0) | 15,315 (82.0) | <0.001 |
| Previous myocardial  infarction, n (%) | 1,673 (9.0) | 1,672 (8.9) | <0.001 |
| Congestive heart failure, n (%) | 9,835 (52.7) | 9,832 (52.6) | 0.001 |
| Peripheral vascular disease, n (%) | 990 (5.3) | 992 (5.3) | <0.001 |
| Cerebrovascular disease, n (%) | 5,498 (29.4) | 5,503 (29.5) | <0.001 |
| Dementia, n (%) | 3,228 (17.3) | 3,233 (17.3) | 0.001 |
| Institutionalized, n (%) | 2,668 (14.3) | 2,670 (14.3) | <0.001 |
| Non-independent  functional status, n (%) | 6,183 (33.1) | 6,175 (33.1) | 0.001 |
| COPD, n (%) | 4,411 (23.6) | 4,413 (23.6) | <0.001 |
| Current smoker, n (%) | 1,671 (8.9) | 1,668 (8.9) | 0.001 |
| Chronic renal failure, n (%) | 4,389 (23.5) | 4,395 (23.5) | 0.001 |
| Diabetes mellitus, n (%) | 14,526 (77.8) | 14,529 (77.8) | <0.001 |
| Cirrhosis, n (%) | 371 (2.0) | 368 (2.0) | 0.001 |
| Coagulopathy, n (%) | 1,660 (8.9) | 1,655 (8.9) | 0.001 |
| Currently receiving  chemotherapy for cancer, n (%) | 163 (0.9) | 161 (0.9) | 0.001 |
| Metastatic cancer, n (%) | 267 (1.4) | 265 (1.4) | 0.001 |
| Drug use disorder, n (%) | 172 (0.9) | 171 (0.9) | <0.001 |
| Alcohol use disorder, n (%) | 282 (1.5) | 282 (1.5) | <0.001 |
| Major psychiatric illness, n (%) | 2,444 (13.1) | 2,447 (13.1) | <0.001 |
| Advanced directive  limiting care, n (%) | 2,174 (11.6) | 2,174 (11.6) | <0.001 |
| An ASD <0.1 is considered balanced.  *RCRI, Revised Cardiac Risk Index; ASD, Absolute Standardized Difference; AIS, Abbreviated Injury Scale; COPD, Chronic Obstructive Pulmonary Disease* | | | |

| **Supplemental Table S9.** Demographic and clinical characteristics of hip fracture patients with RCRI 3, after inverse probability weighting | | | |
| --- | --- | --- | --- |
|  | **Surgery ≤24 hours (N = 3,557)** | **Surgery >24 hours (N = 3,561)** | **ASD** |
| Age, median [IQR] | 78 [72.0-84.0] | 78 [72.0-83.0] | 0.001 |
| Sex, n (%) |  |  | 0.003 |
| Female | 1,860 (52.3) | 1,856 (52.1) |  |
| Male | 1,697 (47.7) | 1,705 (47.9) |  |
| Race, n (%) |  |  |  |
| White | 2,981 (83.8) | 2,983 (83.8) | 0.002 |
| Black | 335 (9.4) | 339 (9.5) | 0.004 |
| Asian | 40 (1.1) | 38 (1.1) | 0.005 |
| American Indian | 25 (0.7) | 25 (0.7) | <0.001 |
| Pacific Islander | 11 (0.3) | 10 (0.3) | 0.003 |
| Other | 158 (4.4) | 159 (4.5) | 0.001 |
| Head AIS, n (%) |  |  | <0.001 |
| Injury not present | 3,411 (95.9) | 3,416 (95.9) |  |
| 1 | 145 (4.1) | 145 (4.1) |  |
| Face AIS, n (%) |  |  | 0.001 |
| Injury not present | 3,442 (96.8) | 3,447 (96.8) |  |
| 1 | 114 (3.2) | 114 (3.2) |  |
| Neck AIS, n (%) |  |  | 0.001 |
| Injury not present | 3,552 (99.9) | 3,556 (99.9) |  |
| 1 | 5 (0.1) | 5 (0.1) |  |
| Spine AIS, n (%) |  |  | 0.002 |
| Injury not present | 3,552 (99.9) | 3,556 (99.9) |  |
| 1 | 4 (0.1) | 5 (0.1) |  |
| Thorax AIS, n (%) |  |  | 0.001 |
| Injury not present | 3,519 (98.9) | 3,523 (98.9) |  |
| 1 | 38 (1.1) | 38 (1.1) |  |
| Abdomen AIS, n (%) |  |  | 0.013 |
| Injury not present | 3,536 (99.4) | 3,536 (99.3) |  |
| 1 | 21 (0.6) | 25 (0.7) |  |
| Upper extremity AIS, n (%) |  |  | 0.002 |
| Injury not present | 3,255 (91.5) | 3,257 (91.5) |  |
| 1 | 302 (8.5) | 304 (8.5) |  |
| Lower extremity AIS, n (%) |  |  | <0.001 |
| 3 | 3,557 (100.0) | 3,561 (100.0) |  |
| 4 | 0 (0.0) | 0 (0.0) |  |
| 5 | 0 (0.0) | 0 (0.0) |  |
| External AIS, n (%) |  |  | 0.001 |
| Injury not present | 3,518 (98.9) | 3,522 (98.9) |  |
| 1 | 39 (1.1) | 39 (1.1) |  |
| Type of fracture, n (%) |  |  | 0.005 |
| Cervical | 1,315 (37.0) | 1,319 (37.0) |  |
| Basicervical | 194 (5.4) | 192 (5.4) |  |
| Pertrochanteric | 1,905 (53.6) | 1,910 (53.6) |  |
| Subtrochanteric | 143 (4.0) | 140 (3.9) |  |
| Type of surgery, n (%) |  |  | 0.001 |
| Internal fixation | 2,443 (68.7) | 2,444 (68.6) |  |
| Arthroplasty | 1,113 (31.3) | 1,117 (31.4) |  |
| Hypertension, n (%) | 2,903 (81.6) | 2,908 (81.7) | 0.001 |
| Previous myocardial  infarction, n (%) | 783 (22.0) | 783 (22.0) | <0.001 |
| Congestive heart failure, n (%) | 2,793 (78.5) | 2,796 (78.5) | <0.001 |
| Peripheral vascular disease, n (%) | 284 (8.0) | 286 (8.0) | 0.002 |
| Cerebrovascular disease, n (%) | 1,527 (42.9) | 1,533 (43.0) | 0.002 |
| Dementia, n (%) | 586 (16.5) | 590 (16.6) | 0.002 |
| Institutionalized, n (%) | 517 (14.5) | 523 (14.7) | 0.005 |
| Non-independent  functional status, n (%) | 1,257 (35.3) | 1,262 (35.4) | 0.002 |
| COPD, n (%) | 927 (26.1) | 928 (26.1) | <0.001 |
| Current smoker, n (%) | 288 (8.1) | 290 (8.2) | 0.002 |
| Chronic renal failure, n (%) | 1,862 (52.4) | 1,868 (52.5) | 0.002 |
| Diabetes mellitus, n (%) | 3,156 (88.7) | 3,157 (88.7) | 0.002 |
| Cirrhosis, n (%) | 89 (2.5) | 92 (2.6) | 0.004 |
| Coagulopathy, n (%) | 368 (10.3) | 363 (10.2) | 0.005 |
| Currently receiving  chemotherapy for cancer, n (%) | 20 (0.6) | 20 (0.6) | 0.002 |
| Metastatic cancer, n (%) | 59 (1.7) | 56 (1.6) | 0.007 |
| Drug use disorder, n (%) | 40 (1.1) | 42 (1.2) | 0.003 |
| Alcohol use disorder, n (%) | 56 (1.6) | 57 (1.6) | 0.003 |
| Major psychiatric illness, n (%) | 512 (14.4) | 511 (14.3) | 0.002 |
| Advanced directive  limiting care, n (%) | 436 (12.3) | 440 (12.4) | 0.003 |
| An ASD <0.1 is considered balanced.  *RCRI, Revised Cardiac Risk Index; ASD, Absolute Standardized Difference; AIS, Abbreviated Injury Scale; COPD, Chronic Obstructive Pulmonary Disease* | | | |

| **Supplemental Table S10.** Demographic and clinical characteristics of hip fracture patients with RCRI ≥4, after inverse probability weighting | | | |
| --- | --- | --- | --- |
|  | **Surgery ≤24 hours (N = 494)** | **Surgery >24 hours (N = 486)** | **ASD** |
| Age, median [IQR] | 77 [72.0-82.0] | 77 [72.0-84.0] | 0.002 |
| Sex, n (%) |  |  | 0.013 |
| Female | 235 (47.5) | 228 (46.9) |  |
| Male | 259 (52.5) | 258 (53.1) |  |
| Race, n (%) |  |  |  |
| White | 395 (80.0) | 387 (79.7) | 0.006 |
| Black | 49 (9.9) | 50 (10.3) | 0.013 |
| Asian | 16 (3.2) | 14 (3.0) | 0.011 |
| American Indian | 4 (0.8) | 3 (0.7) | 0.013 |
| Pacific Islander | 3 (0.6) | 3 (0.6) | 0.005 |
| Other | 28 (5.6) | 29 (5.9) | 0.014 |
| Head AIS, n (%) |  |  | 0.001 |
| Injury not present | 481 (97.4) | 473 (97.4) |  |
| 1 | 13 (2.6) | 13 (2.6) |  |
| Face AIS, n (%) |  |  | 0.019 |
| Injury not present | 480 (97.2) | 473 (97.5) |  |
| 1 | 14 (2.8) | 12 (2.5) |  |
| Neck AIS, n (%) |  |  | <0.001 |
| Injury not present | 494 (100.0) | 486 (100.0) |  |
| 1 | 0 (0.0) | 0 (0.0) |  |
| Spine AIS, n (%) |  |  | <0.001 |
| Injury not present | 494 (100.0) | 486 (100.0) |  |
| 1 | 0 (0.0) | 0 (0.0) |  |
| Thorax AIS, n (%) |  |  | 0.041 |
| Injury not present | 484 (97.8) | 478 (98.4) |  |
| 1 | 11 (2.2) | 8 (1.6) |  |
| Abdomen AIS, n (%) |  |  | 0.090 |
| Injury not present | 492 (99.6) | 486 (100.0) |  |
| 1 | 2 (0.4) | 0 (0.0) |  |
| Upper extremity AIS, n (%) |  |  | 0.004 |
| Injury not present | 456 (92.2) | 448 (92.3) |  |
| 1 | 38 (7.8) | 37 (7.7) |  |
| Lower extremity AIS, n (%) |  |  | <0.001 |
| 3 | 494 (100.0) | 486 (100.0) |  |
| 4 | 0 (0.0) | 0 (0.0) |  |
| 5 | 0 (0.0) | 0 (0.0) |  |
| External AIS, n (%) |  |  | 0.001 |
| Injury not present | 488 (98.8) | 480 (98.8) |  |
| 1 | 6 (1.2) | 6 (1.2) |  |
| Type of fracture, n (%) |  |  | 0.014 |
| Cervical | 181 (36.6) | 178 (36.7) |  |
| Basicervical | 18 (3.8) | 19 (4.0) |  |
| Pertrochanteric | 273 (55.2) | 266 (54.8) |  |
| Subtrochanteric | 22 (4.4) | 22 (4.5) |  |
| Type of surgery, n (%) |  |  | 0.001 |
| Internal fixation | 345 (69.7) | 338 (69.7) |  |
| Arthroplasty | 150 (30.3) | 147 (30.3) |  |
| Hypertension, n (%) | 413 (83.5) | 406 (83.7) | 0.005 |
| Previous myocardial  infarction, n (%) | 234 (47.4) | 238 (48.9) | 0.030 |
| Congestive heart failure, n (%) | 456 (92.1) | 447 (92.1) | 0.003 |
| Peripheral vascular disease, n (%) | 64 (12.9) | 65 (13.4) | 0.014 |
| Cerebrovascular disease, n (%) | 345 (69.7) | 334 (68.8) | 0.020 |
| Dementia, n (%) | 74 (15.0) | 70 (14.5) | 0.015 |
| Institutionalized, n (%) | 85 (17.2) | 80 (16.4) | 0.023 |
| Non-independent  functional status, n (%) | 185 (37.4) | 178 (36.6) | 0.016 |
| COPD, n (%) | 158 (32.0) | 157 (32.3) | 0.006 |
| Current smoker, n (%) | 58 (11.8) | 57 (11.8) | 0.002 |
| Chronic renal failure, n (%) | 353 (71.3) | 347 (71.5) | 0.003 |
| Diabetes mellitus, n (%) | 469 (95.0) | 459 (94.5) | 0.022 |
| Cirrhosis, n (%) | 11 (2.3) | 12 (2.5) | 0.014 |
| Coagulopathy, n (%) | 52 (10.6) | 54 (11.0) | 0.015 |
| Currently receiving  chemotherapy for cancer, n (%) | 7 (1.4) | 7 (1.4) | 0.001 |
| Metastatic cancer, n (%) | 6 (1.2) | 7 (1.4) | 0.009 |
| Drug use disorder, n (%) | 7 (1.4) | 6 (1.2) | 0.014 |
| Alcohol use disorder, n (%) | 6 (1.3) | 7 (1.4) | 0.015 |
| Major psychiatric illness, n (%) | 66 (13.3) | 63 (13.0) | 0.010 |
| Advanced directive  limiting care, n (%) | 78 (15.7) | 71 (14.5) | 0.034 |
| An ASD <0.1 is considered balanced.  *RCRI, Revised Cardiac Risk Index; ASD, Absolute Standardized Difference; AIS, Abbreviated Injury Scale; COPD, Chronic Obstructive Pulmonary Disease* | | | |
